# A Digital Lifestyle Coach (E-Supporter 1.0) to Support People With Type 2 Diabetes: Participatory Development Study

**DOI:** 10.2196/40017

**Published:** 2023-01-12

**Authors:** Eclaire A G Hietbrink, Anouk Middelweerd, Pepijn van Empelen, Katharina Preuhs, Annemieke A J Konijnendijk, Wendy Oude Nijeweme-d’Hollosy, Laura K Schrijver, Gozewijn D Laverman, Miriam M R Vollenbroek-Hutten

**Affiliations:** 1 Department of Biomedical Signals and Systems University of Twente Enschede Netherlands; 2 ZGT Academy Ziekenhuisgroep Twente Almelo Netherlands; 3 Department of Child Health TNO (Netherlands Organization for Applied Scientific Research) Leiden Netherlands; 4 Department of Internal Medicine/Nephrology Ziekenhuisgroep Twente Almelo Netherlands; 5 Board of Directors Medisch Spectrum Twente Enschede Netherlands

**Keywords:** eHealth, mHealth, diet, nutrition, physical activity, lifestyle change, coaching, dynamic tailoring, behavior change, blended care, type 2 diabetes, design, treatment, chronic disease, behavioral, theory, intervention, acceptability, usability, cost

## Abstract

**Background:**

A healthy lifestyle, including regular physical activity and a healthy diet, is becoming increasingly important in the treatment of chronic diseases. eHealth interventions that incorporate behavior change techniques (BCTs) and dynamic tailoring strategies could effectively support a healthy lifestyle. E-Supporter 1.0 is an eCoach designed to support physical activity and a healthy diet in people with type 2 diabetes (T2D).

**Objective:**

This paper aimed to describe the systematic development of E-Supporter 1.0.

**Methods:**

Our systematic design process consisted of 3 phases. The definition phase included the selection of the target group and formulation of intervention objectives, and the identification of behavioral determinants based on which BCTs were selected to apply in the intervention. In the development phase, intervention content was developed by specifying tailoring variables, intervention options, and decision rules. In the last phase, E-Supporter 1.0 integrated in the Diameter app was evaluated using a usability test in 9 people with T2D to assess intervention usage and acceptability.

**Results:**

The main intervention objectives were to stimulate light to moderate-vigorous physical activities or adherence to the Dutch dietary guidelines in people with T2D. The selection of behavioral determinants was informed by the health action process approach and theories explaining behavior maintenance. BCTs were included to address relevant behavioral determinants (eg, action control, self-efficacy, and coping planning). Development of the intervention resulted in 3 types of intervention options, consisting of motivational messages, behavioral feedback, and tailor-made supportive exercises. On the basis of IF-THEN rules, intervention options could be tailored to, among others, type of behavioral goal and (barriers to) goal achievement. Data on these variables could be collected using app data, activity tracker data, and daily ecological momentary assessments. Usability testing revealed that user experiences were predominantly positive, despite some problems in the fixed delivery of content.

**Conclusions:**

The systematic development approach resulted in a theory-based and dynamically tailored eCoach. Future work should focus on expanding intervention content to other chronic diseases and lifestyle behaviors, enhancing the degree of tailoring and evaluating intervention effects on acceptability, use, and cost-effectiveness.

## Introduction

### Background

In 2020, nearly 60% of Dutch adults had experienced one or more chronic diseases [1]. Treatment predominantly focused on drug therapies to make the disease manageable. Although prescribing medication is often an important treatment option, it does not address the fact that chronic diseases can be exacerbated by an unhealthy lifestyle [2,3]. It has been increasingly recognized that a healthy lifestyle, such as a healthy diet, adequate physical activity, and enough sleep, can contribute to a reduction in disease burden, improved quality of life (QoL), and reversal of chronic diseases [4-7]. For example, several studies have shown positive effects of a healthy lifestyle on glycemic control, QoL, medication use, and risk of complications in people with type 2 diabetes (T2D) [8-14]. Furthermore, in people with chronic pulmonary diseases, a healthy lifestyle has shown to improve QoL and to reduce hospitalizations and mortality [15].

Despite the positive effects of healthy lifestyle on the course of chronic diseases, people often find it challenging to live healthy. For instance, >50% of Dutch adults adhered to the Dutch Physical Activity Guidelines [16] and only a quarter of Dutch adults met the Dutch dietary guidelines [17]. Moreover, adherence to healthy lifestyle behaviors appears to be even lower among people with chronic diseases [18-21]. A higher adherence to these guidelines is important for the positive effects of a healthy lifestyle to reach its potential. Realizing sustainable lifestyle improvements requires individuals to self-manage their behavior using a range of skills, such as knowledge acquisition, self-monitoring, action, and coping planning [22]. Developing self-management skills has proven successful in allowing individuals to effectively manage their disease and improve health outcomes [23,24]. However, self-management skills are often insufficient in people with chronic diseases, especially regarding lifestyle behaviors [25-27]. Self-management skills vary from person to person and are subject to various factors (eg, health literacy and socioeconomic status) [28], resulting in each individual needing a personal approach. Hence, interventions with extensive guidance and more motivational strategies fitting the individual’s characteristics and needs are required to achieve sustainable lifestyle behavior change. However, it is very costly and challenging to provide extensive guidance via face-to-face programs alone with the rising capacity issues and limited financial resources available in health care domain [29,30]. Therefore, eHealth (ie, the use of technology to support health, well-being, and health care [31]) can be used to contribute to continuous and affordable lifestyle self-management support for people with chronic diseases [32,33].

eHealth has the potential to support lifestyle self-management [34,35]. Guided eHealth interventions have been shown to be as effective as face-to-face treatment in the short term but are generally more cost-effective [36-38]. Moreover, eHealth enables continuous support in daily life and tailoring of support toward an individual [33,39]. Several reviews and meta-analyses showed that app-based eHealth interventions have the potential to improve physical activity levels [40,41] and adherence to dietary guidelines [42,43], resulting in improved health outcomes, such as perceived fitness, body weight, blood pressure, or glycemic control [42-46]. However, eHealth effectiveness has been shown to differ between interventions [47,48], and positive intervention effects are often not sustained in the long term [49-53].

One source of variability in eHealth effectiveness is differences in the use of behavior change theory [54-56]. Behavior change theories have been developed to explain health behaviors and guide health behavior change based on a variety of factors that individually influence and affect the performance of health behavior (ie, behavioral determinants) [57]. Using theory helps us to identify behavioral determinants of behavior that are relevant to the target by means of an intervention to effectively change behavior. Besides, it enables us to determine which behavior change techniques (BCTs) are most likely to bring about change [58,59]. Including behavior change theory is key as interventions have shown to be more effective when they are theory based [46,60-63]. Moreover, using theories specifically explaining behavioral maintenance, in addition to theories that primarily focus on initial change, may be useful for the design of interventions to achieve sustainable behavior change [64,65].

Systematic reviews showed that tailored eHealth interventions are more effective in promoting healthy behaviors and user engagement than generic interventions [44,47,63,66-68]. The effect sizes of static tailored interventions (ie, coaching based on a single baseline assessment) remain small, whereas dynamically tailored interventions (ie, coaching based on iterative assessment) show larger effect sizes and have long-term effects [69,70]. Smartphones and activity trackers enable us to dynamically tailor interventions [71] to an individual (eg, personal goals and self-efficacy levels) and their environment (eg, location and weather) [72]. Given the positive attitude toward smartphone and technology use by both the young and older adults [73,74], it is assumed that dynamically tailored eHealth interventions provide a great opportunity to facilitate behavior change.

eHealth interventions are expected to be more beneficial when provided in a blended care setting (ie, combining eHealth and regular health care) [75-78]. A previous study has shown that blended care, including personal guidance of patients, leads to a higher and better use of eHealth interventions [75], which can result in improved intervention effects [31]. Moreover, the first studies in this emerging research field showed positive effects on intervention effectiveness [36,79-81]. Besides, it is foreseen that a good integration of eHealth and regular care can lead to higher quality of care and a decrease in consultations with health care professionals [75,82] and can be more cost-effective than regular care [83].

### Objective

In summary, innovative eHealth interventions that are theory based, which include dynamic tailoring, and are offered as blended care may help to effectively support health behavior change. However, eHealth interventions that integrate these potential success factors are scarce to date. Therefore, the aim of this paper is to successively describe the systematic development and usability testing of the first version of E-Supporter, a theory-based, dynamically tailored, and blended lifestyle coaching intervention for people with chronic diseases.

## Methods

### Ethics Approval

Ethics approval was obtained from the Medical Research Ethics Committees United Nieuwegein, the Netherlands (R20.121). Written consent was requested from each patient to participate in the study. All participants gave verbal consent before starting the audio recording of the interviews. Data privacy was protected in accordance with the General Data Protection Regulation standard. Participants were informed in detail about how data were collected, processed, and stored in the subject information sheet. Participants gave explicit consent for the use of their data by signing the informed consent form. Data privacy was protected by offering anonymous preset accounts without personal data to prevent sharing personal data with commercial parties.

### Project Overview

**Figure 1:**
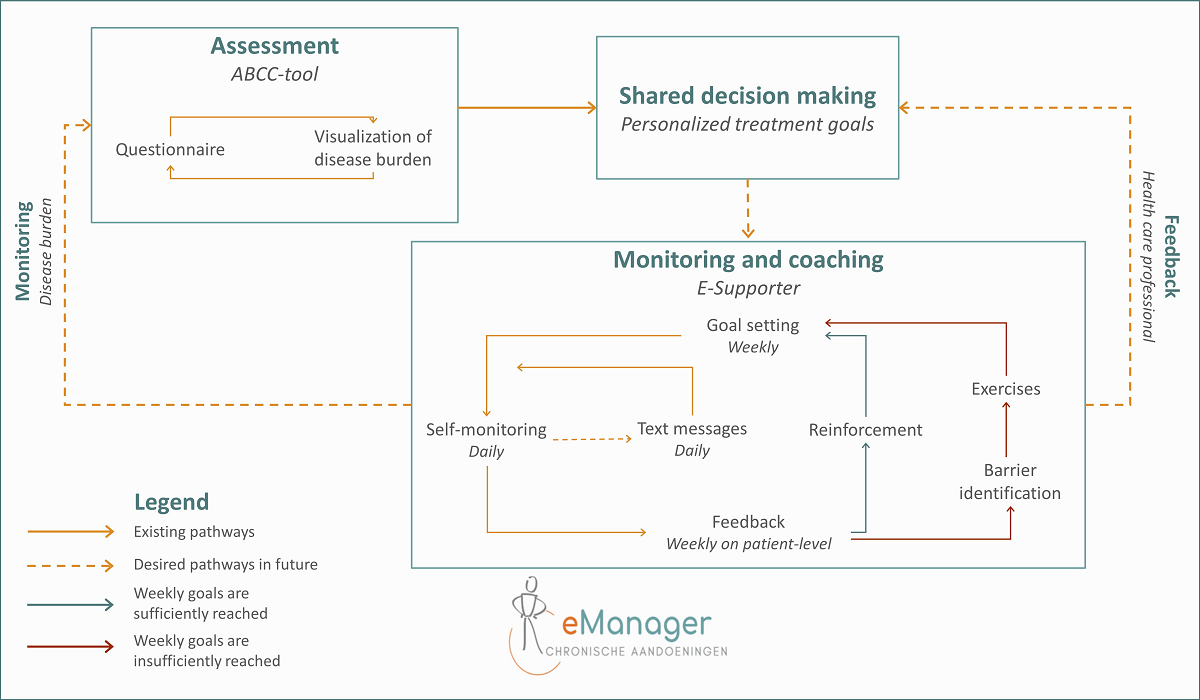
eManager chronic diseases. ABCC: Assessment of Burden of Chronic Conditions.

The development of E-Supporter is part of the eManager project [84], which aims to enhance patient-centered health care to reduce disease burden in the Netherlands. The eManager project focuses on blended coaching, which consists of the Assessment of Burden of Chronic Conditions (ABCC) tool and E-Supporter (Figure 1).

**Figure 2:**
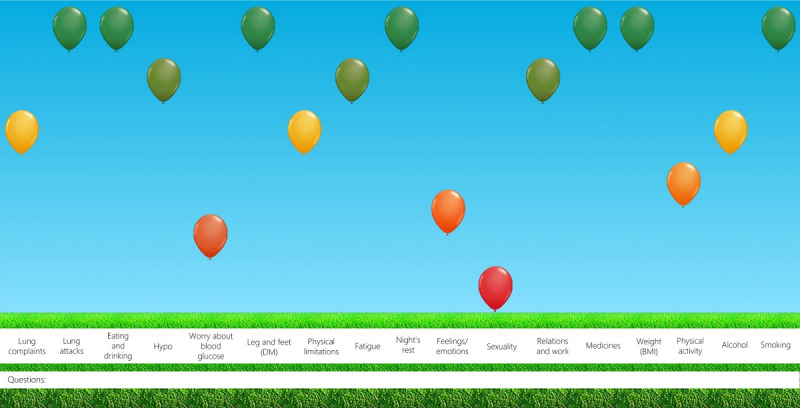
Visualization of disease burden measured by the Assessment of Burden of Chronic Conditions (ABCC) tool [85]. DM: diabetes mellitus.

The ABCC tool is already being used during consultations with a health care professional and is developed for several chronic diseases, including chronic obstructive pulmonary disease, asthma, T2D, and chronic heart failure. The ABCC tool maps the patient’s disease burden based on a questionnaire that the patient completes before consultation and based on medical information from the electronic patient file [85]. During consultation, the burden of disease in various generic and disease-specific domains is clearly presented in a balloon chart (Figure 2), which shows domains that can be improved. The ABCC tool also shows treatment recommendations based on existing guidelines for each domain when selecting a balloon from the image. On the basis of a discussion between the patient and health care professional after the treatment advice, personalized treatment goals can be determined. Detailed information on the development of the ABCC tool can be found elsewhere [85].

E-Supporter aims to support the patient in daily life in pursuing the earlier established personalized treatment goals. Diabetes professionals can offer E-Supporter to patients in a shared decision-making process. E-Supporter focuses on obtaining and maintaining a healthy lifestyle to reduce the perceived burden of disease. In this paper, the development of the first version of E-Supporter (ie, the components as presented within the *Monitoring and Coaching* frame; Figure 1) is described. In future versions, E-Supporter will also be used to guide health care professionals to be better informed about their patients based on the progress that can be monitored by means of E-Supporter.

### Intervention Development Approach

#### Overview

The systematic design of the intervention was guided by program-planning models [39,86], and the design was categorized into 3 phases: (1) a definition phase in which we analyzed the program goals and possible behavior change strategies, (2) a development phase for which we relied on Nahum-Shani’s model [39] for describing the elements of the intervention, and (3) the evaluation phase in which the developed content was evaluated (Textbox 1). Content for specific target groups or lifestyle behaviors can be added step by step by repeating the development process.

We followed a participatory development process by combining the perspectives of 9 health care professionals and 33 people with T2D. The team of health care professionals consisted of 4 internists, 3 diabetes nurses, and 2 physician researchers. The health care professionals contributed during all phases of the development approach at research group meetings and focus groups by formulating intervention objectives, formulating lifestyle advice for people with T2D, and reviewing and revising the motivational message. Multimedia Appendix 1 provides detailed information on the methods for the focus group discussions for assessing and revising the motivational messages.

The requirements for digital lifestyle coaching from the patient’s perspective emerged from interviews with 19 people with T2D. The results of these interviews are described elsewhere [87]. These requirements were translated into content for E-Supporter 1.0. During the development process, subsets of the motivational messages were evaluated in iterations among 14 people with T2D to gain insights into their opinions. The input derived from these iterative evaluations was used to refine and improve the intervention content.

**Description of the stepwise process for the development of E-Supporter.**

- Phase 1: definition
  - Step 1: target population and behaviors
    - Selecting a target group and behaviors to promote in the intervention
  - Step 2: intervention objectives
    - Defining the main and subobjectives of the intervention
  - Step 3: selection of behavioral determinants
    - Selecting relevant behavioral determinants based on existing behavior change models and describing the determinants of behavior to target in the intervention
  - Step 4: selection of behavior change techniques (BCTs)
    - Selecting BCTs to address determinants of behavior based on existing studies and reviews
- Phase 2: development
  - Step 1: tailoring variables
    - Deciding which information concerning the individual will be used for tailoring (ie, to decide when and how to intervene)
  - Step 2: decision rules
    - Operationalizing of intervention options by specifying which intervention option to offer to whom
  - Step 3: intervention options
    - Developing possible actions (ie, interventions) that might be employed during the intervention period
  - Step 4: integration of E-Supporter 1.0
    - Describing the integration of the E-Supporter content in an app for people with type 2 diabetes (T2D)
- Phase 3: evaluation
  - Usability test
    Usability testing of the intervention in a mobile app on acceptability and usage in 9 patients with T2D

#### Phase 1: Definition Phase

The program-planning model developed by Kreuter et al [86,88] provided a basis for the step-by-step plan to define the theoretical framework of the intervention. First, our choice to select people with T2D as the target group and physical activity and nutrition as target behaviors was substantiated. Second, the intervention objectives were determined based on the existing Dutch standards of care for people with T2D and guidelines regarding physical activity and healthy nutrition. Third, the selection of changeable behavioral determinants that needed to be addressed in the intervention was guided by behavioral theories. Finally, there was the need to identify BCTs to influence these determinants. We searched in literature for BCTs that could be linked to a particular determinant or the selected health behaviors.

#### Phase 2: Development Phase

In accordance with Nuham-Shani’s model [39], the requirements from the definition phase were translated into intervention content by defining (1) tailoring variables that comprise the information that is used to decide when and how to intervene, (2) intervention options that are defined as the type of support offered (eg, information, feedback, and advice), and (3) decision rules, including the operationalization of decision points when a particular intervention option is delivered. The last step of the development phase comprised the integration of E-Supporter 1.0 in two mobile health apps: (1) *the Diameter* app [89,90] and (2) *MiGuide* [91]. Both apps provide blended lifestyle support for people with T2D by means of lifestyle monitoring and coaching.

#### Phase 3: Evaluation Phase

After being integrated in the Diameter app, E-Supporter 1.0 was evaluated during a 5-week usability study among 9 people with T2D. The Diameter app was used because its purpose closely aligned with the initial design and application of E-Supporter 1.0 to encourage physical activity and a healthy diet in people with T2D. This study was the first to evaluate the E-Supporter integrated into an app. Therefore, the aim of the study was two-fold: (1) to gain insight into intervention use and acceptability of E-Supporter 1.0 integrated in the Diameter app and (2) to identify technical issues in the integration of E-Supporter 1.0 within the Diameter app. In total, 9 patients with T2D visiting the outpatient clinic at the Ziekenhuis Groep Twente Hospital were recruited; they were ≥18 years and were familiar with an Android smartphone (version 5.0 or higher). Patients were not able to participate when they underwent renal replacement therapy, were engaged in drug abuse, or had insufficient proficiency in the Dutch language. Participants used the Diameter app, with E-Supporter content, in combination with the activity tracker Fitbit Inspire 2 [92] and Freestyle Libre 2 sensor (ie, a continuous glucose monitoring sensor in the interstitial fluid of the upper arm) [93,94] for 5 weeks at home. The Freestyle Libre 2 sensor was one of the self-monitoring tools of the Diameter app to provide continuous insights into glucose values for people with T2D. Participants were asked to use the Diameter app as instructed by the researchers. For this, participants were asked to scan the Freestyle Libre 2 sensor at least 3 times a day (to prevent data loss), to wear the Fitbit activity tracker every day, and to fill in the food diary for at least 6 days [95]. As part of the E-Supporter 1.0 components, participants had the opportunity to set a lifestyle goal, read daily motivational messages, and perform weekly psychological exercises.

A mixed methods approach was used to explore intervention use and acceptability of E-Supporter 1.0 and its integration within the Diameter app. Intervention use was exploratively assessed using log data of the E-Supporter 1.0 components (ie, motivational messages and physiological exercises) and Diameter app components (ie, Fitbit, food diary and Freestyle Libre 2). Use of the Diameter app and E-Supporter components was reported by describing frequency and duration of the used functionalities. Log data were also used to identify whether the intervention was delivered as intended and to identify technical integration issues. Open-ended interviews based on the Unified Theory of Acceptance and Use of Technology 2 model [96] were conducted to capture participants’ experiences with E-Supporter integrated into the Diameter app. Interview topics included the general appreciation of the Diameter; ease of use of the Diameter; perceived usefulness of the Diameter; perceived usefulness of the E-Supporter content; appreciation and perceived enjoyment of the E-Supporter content; technical infrastructure to use the Diameter; and the Diameter in the health care process. The transcripts were coded using inductive thematic analysis [97]. Multimedia Appendix 2 provides more detailed information about the methods of the usability study.

## Results

### Phase 1: Definition Phase

#### Step 1: Target Population and Behaviors

E-Supporter 1.0 focused on improving physical activity and a healthy diet in people with T2D. Diabetes is one of the four major types of noncommunicable diseases worldwide [98]. It is also expected that until 2040 the prevalence of diabetes will rise relatively sharply compared with other diseases. This prospect calls for initiatives to reduce the burden of T2D on patients and the health care system. There is growing evidence that lifestyle interventions can positively contribute to the management of T2D [6]. Many studies showed that lifestyle interventions targeting physical activity or diet can achieve reversion or remission of T2D [8,9,11-13]. The aforementioned studies provide insight into the importance of sufficient physical activity and a healthy diet for improved glycemic regulation, reduction of medication use, and possible reversal of T2D. However, a substantial proportion of people with T2D do not meet physical activity and eating guidelines [21,99-103]. To illustrate, previous studies found that adherence to Dutch Healthy Diet Guidelines was low among participants with T2D [102]. In another example, more than one-third of the participants with T2D had limited physical activity (ie, <5000 steps per day) [103]. Therefore, it is important to improve these behaviors among people with T2D so that they can benefit from the positive effects on their health.

#### Step 2: Intervention Objectives

##### Physical Activity

The Dutch health care standard for T2D states that small improvements in physical activity levels can already lead to positive health effects, although being physically active for longer periods more often and more intensively does have additional health benefits [104]. The Dutch Health Council emphasizes the aim to achieve the following physical activity levels for the general population: 150 minutes (about 2.5 hours) per week of moderate to vigorous physical activity spread over several days, muscle- and bone-strengthening activities at least twice a week, and to minimize sitting hours [105]. Moreover, it is stated that advice should be aligned with the motivation, possibilities, and daily routine of the person with T2D. Current physical activity guidelines make no statements about light-intensity physical activities (ie, activities that are classified as >1.5 to <3 metabolic equivalents [106], such as slow walking, shopping, or household chores). However, recent studies have shown that light physical activities are beneficially associated with health outcomes [107,108]. People may be more inclined to replace physical inactivity with light-intensity physical activities, which are usually easier to incorporate into daily life [109]. Therefore, the primary aim was to facilitate small step-by-step improvements or maintenance of light to moderate-vigorous physical activities in people with T2D, aligning with their motivation, possibilities, and daily routine.

##### Nutrition

The Dutch *Guidelines for a good diet* from 2015 were used to develop the nutritional module of E-Supporter [104,110]. The Dutch Health Council states that essential elements for a healthy diet for people with T2D are already part of the national guidelines, such as reducing the consumption of unhealthy carbohydrate-rich foods (eg, refined grain products). In line with the Dutch Health Council’s recommendations, the main goal of the nutritional module was to increase adherence to the Dutch dietary guidelines, again matching the needs of the individuals closely.

#### Step 3: Selection of Behavioral Determinants

**Figure 3:**
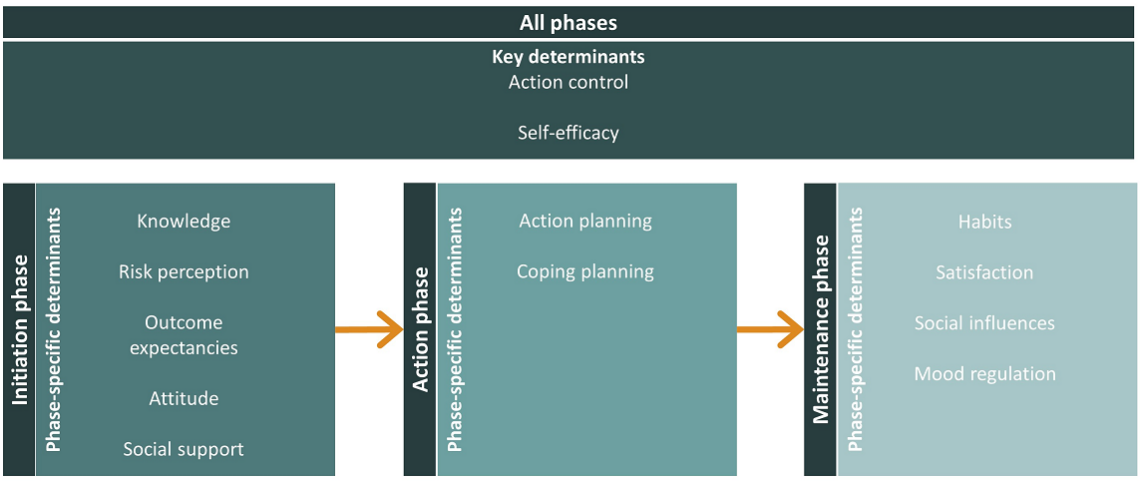
Intervention targets of E-Supporter 1.0.

The health action process approach (HAPA) [111] and theories that elucidate behavioral maintenance (eg, Rothman’s theory of maintenance [112,113] or Marlatt’s relapse prevention theory [114]) were used as a basis for E-Supporter 1.0. The HAPA model distinguishes between a preintentional motivation phase and a postintentional volition phase each with different behavioral determinants [111]. The HAPA model has shown to be able to explain several health behaviors, including physical activity and dietary behaviors. The volitional phase in the HAPA model comprises both action initiation and maintenance [115]; so, it does not include a separate phase to address behavior maintenance. Because health behavior change is often not maintained in the long term [49-53], addressing behavioral maintenance as well after initial change is emphasized [112]. Behavioral maintenance theories explicitly address determinants important for maintenance of behavior in the long term, such as the formation of habits and the perceived value of a new behavior [113]. Because these theories suggest that different behavioral determinants contribute to behavioral initiation and maintenance, it can be argued that separate determinants targeting behavior maintenance could also be included in the intervention approach. As a result, we developed an intervention approach that recognizes three distinct phases of behavior change: (1) an initiation phase to form intentions to adopt a healthy behavior, (2) an action phase to transform intentions into actual behavior change, and (3) a maintenance phase to support persistence of behavior change in the long term (Figure 3). Determinants of behavior were extracted from the HAPA model and behavior maintenance theories. These determinants were addressed in all phases (ie, key determinants) or in one of the three behavioral phases (ie, phase-specific determinants).

In total, 2 key determinants were selected that recur in all phases of the intervention: action control and self-efficacy. Action control comprises three self-regulatory processes: (1) awareness of standards (ie, a self-set goal), (2) self-monitoring that yields information about the attainment of individual’s behavior or goal, and (3) self-regulatory effort to achieve the goal [116,117]. Self-efficacy refers to an individual’s belief in his or her own capability to perform a certain behavior needed to achieve a desired outcome [118]. Self-efficacy is found to be related to the intention to change [119], goal level and goal achievement, and affective reactions, which have an impact on self-regulatory processes that subsequently influence performance of the target behavior [120]. In the initiation phase, intervention options focus primarily on confidence in one’s own capacity to perform the desired behavior (ie, task self-efficacy). Later in the process, this focus shifts to confidence in one’s own capacity to deal with barriers (ie, maintenance self-efficacy) or to recover from setbacks (ie, recovery self-efficacy) [111].

Phase-specific determinants were derived for each of the defined phases of behavior change (Figure 3). To form intentions for behavior change, the determinants risk perception, outcome expectancies, attitude, and social support were selected from the HAPA model and Rothman’s theory. Moreover, knowledge about healthy behavior within the target group is insufficient [121-123] but is required to achieve self-management to realize lifestyle changes [124]. Therefore, the initiation phase was supplemented with the determinant knowledge, for example, by providing general information about guidelines for physical activity and healthy nutrition. Action planning and coping planning were extracted from the HAPA model as phase-specific determinants in the action phase to translate intention into behavior. For the maintenance phase, we focused on the determinants habits, satisfaction, social influences, and mood regulation. The importance of forming habits and satisfaction with behavior were derived from Rothman’s theory of behavior maintenance. In addition, several habit theories emphasize the role of social influences on behavior maintenance [65]. Social influences can increase an individual’s capacity to maintain behavior because social influences can affect an individual’s opinions, emotional states, and behaviors in the long term. Finally, mood regulation was targeted in our intervention. Marlatt and Gordon [114,125] argued that relapse prevention is an important part of coping planning that refers to not only behavioral adaptation but also mood management or repair in case of a behavioral lapse. An overview of the definitions of all determinants can be found in Multimedia Appendix 3 [126-135].

#### Step 4: Selection of BCTs

The selected BCTs per determinant based on the literature search can be found in Table 1.

We covered 3 BCTs identified by Abraham and Michie that address self-regulatory processes from the concept of negative feedback control [59,116], including *review of goals*, *feedback on behavior*, and *self-monitoring of behavior*. Several studies provided evidence for the effectiveness of BCTs that address self-regulatory processes in changing health behavior [136-139]. To illustrate, Michie et al [139] found that interventions that included *self-monitoring of behavior* in combination with at least one other self-regulatory technique (eg, *goal setting*, *feedback on behavior*, or *review of behavioral goals*) were significantly more effective in promoting physical activity and healthy eating compared with interventions which did not include these techniques. Regarding self-efficacy, we incorporated BCTs recognized as effective to increase self-efficacy in literature. Systematic reviews and meta-analyses [140,141] found that several BCTs were significantly associated with improvements in self-efficacy levels and positive changes in physical activity. Of these BCTs, *action planning*, *social support*, and *instruction on how to perform the behavior* were included in the intervention. Furthermore, BCTs were derived to target self-efficacy from 2 studies that identified effective BCTs for several prominent determinants of behavior. First, Kok et al [142] described BCTs that target determinants of behavior based on literature synthesis. Second, Johnston et al [143] linked BCTs to determinants of behavior through the triangulation of findings from literature synthesis and expert consensus. The following BCTs were selected from the aforementioned studies to target self-efficacy levels: *problem solving*, *verbal persuasion about capabilities*, *focus on past success*, *reduce negative emotions*, *goal setting*, and *self-monitoring of behavior*.

**Table 1:** Determinants and linked behavior change techniques incorporated in E-Supporter 1.0.

	Action control	Self-efficacy	Knowledge	Risk perception	Outcome expectations	Attitude	Social support	Action planning	Coping planning	Mood management	Habits	Satisfaction	Social influences
1.1 Goal-setting (behavior)		✓											
1.2 Problem-solving		✓						✓	✓		✓		
1.4 Action planning		✓						✓	✓		✓		
1.5 Review behavior goal	✓												
2.2 Feedback on behavior	✓												
2.3 Self-monitoring of behavior	✓	✓											
2.4 Self-monitoring of outcomes of behavior												✓	
3.1 Social support, including motivational interviewing		✓					✓						✓
3.2 Social support (practical)							✓						✓
4.1 Instruction on how to perform the behavior		✓	✓										
5.1 Information about health consequences			✓	✓	✓	✓							
5.6 Information about emotional consequences					✓								
6.3 Information about others’ approval													✓
7.1 Prompts and cues								✓			✓		
8.3 Habit formation											✓		
9.1 Credible source						✓							
9.2 Pros and cons					✓	✓							
9.3 Comparative imagining of future outcomes					✓								
11.2 Reduce negative emotions		✓								✓			
13.2 Framing and reframing						✓							
15.1 Verbal persuasion about capability		✓											
15.3 Focus on past success		✓											

BCTs that previously were shown to be effective in influencing specific determinants of behavior were extracted from several studies [59,61,142-144] for each of the phase-specific determinants targeted in the intervention (Table 1). For example, the BCT *information about health consequences* can be applied to influence the determinants knowledge, risk perception, outcome expectancies, and attitude toward the behavior [142,143]. In addition, it is worth noting that many of the selected BCTs were found to be effective in promoting health behaviors [139,145-149] and may be associated with improvements in HbA_1c_ among people with T2D [146]. For instance, research found that mainly the combination of action planning and coping planning techniques was effective in improving physical activity levels [147]. In another study, physical activity interventions that included the following BCTs showed larger effect sizes at follow-up (ie, maximum of 6 months) than interventions that did not: *action planning*, *instruction on how to perform the behavior*, and *prompts and cues* [148].

### Phase 2: Development Process

#### Overview

We specified the main intervention features for E-Supporter, consisting of (1) goal-setting options (ie, step goals, cycling goals, or nutritional goals) and (2) intervention options consisting of motivational messages, feedback, and reinforcement or barrier identification combined with psychological exercises (Figure 1). To provide insight into and feedback on current lifestyle behavior, the E-Supporter content can be used in combination with self-monitoring tools (eg, Fitbit activity tracker [150], self-reported activities, and digital food diary) of the apps in which E-Supporter is integrated. On the basis of these main intervention features and the results of the definition phase, we determined the tailoring variables, wrote the decision rules, and developed content for the intervention options (Table 2).

**Table 2:** Examples of workflow E-Supporter 1.0 per tailoring variable.

Tailoring variables	Decision rule	Decision point	Intervention options	Example intervention option
Motivational message based on *duration of intervention use*	*IF* duration ≤15 days *THEN* (initiation phase message) *OR* (phase generic message) *ELSE IF* duration >15 days and duration ≤30 days *THEN* (initiation phase message) *OR* (action phase message) *OR* (phase generic message) *ELSE IF* duration >30 days and duration ≤45 days *THEN* (action phase message) *OR* (phase generic message) *ELSE IF* duration >45 days and duration ≤60 days *THEN* (action phase message) *OR* (maintenance phase message) *OR* (phase generic message) *ELSE IF* duration >60 days *THEN* (maintenance phase message) *OR* (phase generic message)	Twice a day	Informative, motivational, or advisory motivational message	Action phase, disease-generic, morning-specific, and physical activity goal: “Good morning *[name]*, did you know that your environment can encourage you to be more physically active? For example, put your walking shoes by the door. Then you will be reminded to walk!”
Motivational message based on *type of chronic disease*	*IF* type of illness=diabetes *THEN* (diabetes-specific message) *OR* (disease-generic message) *ELSE IF* type of illness=other *THEN* (generic message)	Twice a day	Informative, motivational, or advisory motivational message	Initiation phase, diabetes-specific, any time of the day, and physical activity goal: “Hi *[name]*, Did you know that physical activity reduces the risk of additional physical consequences of diabetes (complications)? Examples of these complications include damage to the feet, nerves, eyes, kidneys and heart and blood vessels.”
Motivational message based on *time of day*	*IF* time ≥9 AM and time ≤12 PM *THEN* (morning-specific message) *OR* (all moments message) *ELSE IF* time >12 PM and time ≤6 PM *THEN* (afternoon specific message) *OR* (all moments message) *ELSE IF* time >6 PM and time ≤9 PM *THEN* (evening specific message) *OR* (all moments message)	Twice a day	Informative, motivational, or advisory motivational message	Initiation phase, morning-specific, diabetes-specific, and nutrition goal: “Eating breakfast is particularly important. People with diabetes who skip breakfast are on average heavier and have higher blood sugar levels. Your body produces less insulin if you do not eat breakfast.”
Motivational message based on *type of behavioral goal*	*IF* behavioral goal=(physical activity) *THEN* (physical activity message) *OR* (all goal message) *ELSE IF* behavioral goal=(nutrition) *THEN* (nutrition message) *OR* (all goal message)	Twice a day	Informative, motivational, or advisory motivational message	Maintenance phase, physical activity, disease generic, and any time of the day: “Hi *[name]*, Is it time to expand your goal? For example, do you now walk 20 minutes on Monday and Wednesday? Then try to walk for 30 minutes on those days. Or you can go for an extra walk, for example on Friday.”
Feedback based on *goal achievement* (step goal example)	*IF* total number of steps≥goal set *THEN* (motivational feedback) *ELSE IF* total number of steps<goal set *THEN* (feedback) *AND* (identify barriers) *AND* (recommend intervention)	7 days after setting a goal	Provision of feedback on goal achievement and examination of barriers and recommendation of an exercise	Goal not achieved: “Hi *[name]*, let us look back at how it went last week. You achieved your goal in 3 out of 7 days last week. It seems that you are currently finding it difficult to reach your goal. Why do you find it difficult to achieve your goal?”
Exercise based on *identified barrier*	*IF* identified barrier=motivation *THEN* (motivation exercise) *ELSE IF* identified barrier=competence *THEN* (self-efficacy exercise) *ELSE IF* identified barrier=mood *THEN* (mood exercise) *ELSE IF* identified barrier=stress *THEN* (stress exercise) *ELSE IF* identified barrier=planning *THEN* (planning exercise) *ELSE THEN* (provide nothing)	7 days after setting a goal	Provision of matching psychological exercise	Mood: “By reflecting on pleasant things that you experience, you become happier. Think of 3 fun things that happened to you today or yesterday. You experienced 3 things that make you happier. If you want, you can make such a list at the end of every day for the next week. Hopefully, this helps you to think positively.”

#### Step 1: Tailoring Variables

In E-Supporter, 6 tailoring variables were applied: duration of intervention use, type of chronic disease, time of day, type of behavior goal, goal achievement, and the identified barrier toward goal achievement.

The first tailoring variable was the *duration of intervention use*. The duration of use of the intervention was measured by calendar after first login and was used to select an intervention option fitting a specific behavior change phase over time (Figure 3). In addition, intervention options were tailored to the *type of chronic disease* because people are more likely to follow advice when it is relevant to them [39]. Therefore, intervention options could be tailored to people with T2D or consisted of generic information related to changing health behaviors. This allows tailoring of information to other diseases in subsequent intervention versions. Another tailoring variable comprised the *time of day.* To better match advice with the time of day, some intervention options contained information appropriate for a particular time of day, namely, in the morning, afternoon, or evening. Other intervention options could be sent at all parts of the day. Time of the day was measured with the smartphone clock. Furthermore, *type of behavior goal* was used as a tailoring variable*.* Intervention options were tailored to the type of behavior that an individual wanted to improve (ie, either physical activity or nutrition) based on the type of weekly goal that was set (ie, step goal, cycling goal, or nutritional goal). *Goal achievement* was also used by tailoring intervention options to the percentage of days per week the behavioral goal was met. Monitoring of goal achievement was based on passive assessment by a Fitbit activity tracker or self-reported goal achievement by means of daily ecological momentary assessments (EMAs) with 24-hour recall. EMA includes repeated sampling of individuals’ current behaviors in real time and in subjects’ natural environments [151]. In addition, *goal achievement* determined whether individuals were asked about promoting factors or barriers for goal achievement and if an intervention option should be delivered (Figure 1). Finally, the *identified barrier toward goal achievement* was used to tailor intervention options and was assessed weekly by EMA.

#### Step 2: Decision Rules

The decision rules were operationalized to provide the right type of intervention option tailored to the user circumstances. The decision rules were based on IF-THEN rules specifying the situation (IF) with the cutoff point of a given situation (eg, if a goal is reached or not) and the characteristics of an intervention option (THEN). There were three types of decision rules: (1) rules that triggered the type of motivational message, (2) rules that triggered feedback on goal achievement, and (3) rules that triggered a type of psychological exercise. Table 2 shows examples of decision rules for each intervention option.

#### Step 3: Intervention Options

##### Motivational Messages

Motivational messages were designed for one-way communication, delivered as push notifications, and written in the Dutch language at the Common European Framework of Reference for Languages B1-level [152]. We developed a database of 425 motivational messages, consisting of content for each of the tailoring variables. Decision points took place at 2 semirandom times per day for a period of 10 weeks.

To be able to tailor motivational messages to the *duration of intervention use*, a set of motivational messages was written for each phase of behavior change. Message content was based on the determinants and corresponding BCTs identified in the definition phase (Table 1). Each BCT was operationalized using the definitions and examples provided in the Behavior Change Technique Taxonomy, version 1. Examples of motivational messages for each BCT can be found in Multimedia Appendix 3. Addressing the different phases of behavior change over time was reflected as follows: (1) earlier messages focused more explicitly on persuasive messages (targeting attitudes) or awareness raising (targeting knowledge and risk perceptions), (2) later messages focused more on performing new behavior (targeting action and coping planning), and (3) the latest messages focused on behavioral maintenance (eg, targeting habit formation). In addition, the key determinants were addressed throughout the whole duration of the intervention. For the tailoring variable *type of chronic disease*, content of the messages was divided into advice that applied to everyone (ie, generic messages) and advice that only applied to people with T2D (ie, diabetes-specific messages). Motivational messages that aimed at a specific *moment of the day* contained information appropriate to that time of day. For example, messages about breakfast were sent in the morning or about taking a lunch walk in the afternoon. To tailor messages to the *type of behavior goal,* we developed 3 types of messages: goal independent messages, physical activity messages, and nutritional messages. The content of the messages was aligned with the intervention objectives. For example, motivational messages for physical activity mainly focused on promoting light to moderate physical activities that were considered as most feasible for the target group, such as gardening, brisk walking, and cycling [109]. For this, health information was used from books, reliable websites (eg, the website of the Dutch Nutrition Center), national lifestyle guidelines, and diabetes specialists.

During the focus groups with health care professionals, 74.6% (208/279) of messages were directly approved. Furthermore, 20.1% (56/279) of motivational messages were revised by reformulating texts and were approved afterward. In addition, 5.4% (15/279) of messages were excluded from the database. The main reasons for adaptation or exclusion were that messages (1) were too difficult to understand, (2) contained information that only health care professionals are allowed to give, and (3) raised unrealistic expectations of the effects of a healthy lifestyle.

##### Feedback

Feedback based on *goal achievement* was provided weekly after the last goal assessment moment of the week. Feedback was given regarding whether they achieved their goal, consisting of both descriptive and evaluative feedback. Everyone received feedback on how many days the goal was achieved in the past week. If individuals realized their goal substantially or even their full goal, they received a compliment (eg, “You’re doing well!”). In addition, the users were prompted about promoting factors by asking “Think about what helped you to work on and achieve your goal this week” and “Is this something which might help you next week as well?” (Figure 1). If individuals had limited achievement of their goal, they received feedback such as “At the moment it seems difficult to achieve your goal. What is the main reason for this?” Thereafter, motivation, self-efficacy, mood, stress, or planning problems were assessed to identify barriers to goal achievement.

##### Psychological Exercises

When a goal was not sufficiently reached and motivation, self-efficacy, mood, stress, or planning was the *identified barrier* for goal achievement, an appropriate psychological exercise (fitting the indicated barrier) option was selected to support problem-solving. If participants realized their weekly goal sufficiently, they were offered an exercise of choice (eg, self-efficacy), after being stimulated to think about promoting factors that helped them in their goal progress. In both cases, individuals were able to decline the exercise and received good luck wishes for the coming week. Whenever individuals agreed to complete an exercise, a random exercise concerning the chosen determinant was initiated. In total, there were 5 distinct categories of behavioral determinants with varying amounts of related exercises: motivation (n=3), self-efficacy (n=5), planning (n=4), mood (n=8), and stress (n=2). An overview of exercises and related content per determinant can be found in Multimedia Appendix 4.

Exercises comprised a dialog between the user and conversational agent using motivational interviewing techniques (Table 1; BCT 3.1), which is a direct, person-centered conversation style that promotes behavioral change by strengthening an individual’s intrinsic motivation and commitment to change [153]. During a dialog, the user was prompted to think, plan, or elaborate on setting future steps toward the health behavior and reply to questions of the coach by completing open input fields or choosing a predefined answer fitting the user’s response. The response of the coach was selected from a set of possible predefined answers depending on the user’s input. For example, in the exercise *importance ruler* related to the determinant motivation, users were asked to indicate how important the health behavior was for them and why they think it was important to them. At the end of the exercise, the user received a summary highlighting why engaging in the respective health behavior was important to them. Finally, all users, independent of goal achievement, received the possibility to adapt their weekly goal. Goal setting was guided by the coach and consisted of three options, including (1) preserve the current goal, (2) adapt the current goal (ie, increasing or decreasing the difficulty of goal), or (3) setting a new goal (eg, from step goal to nutritional goal).

#### Step 4: Integration of the E-Supporter 1.0

The content of E-Supporter 1.0 was (partly) integrated into two apps: (1) *the Diameter* [89,90] and (2) *MiGuide* [91]. Both apps aim to monitor and coach people with T2D and encourage the adoption of a healthy lifestyle.

##### Integration Within the Diameter App

**Figure 4:**
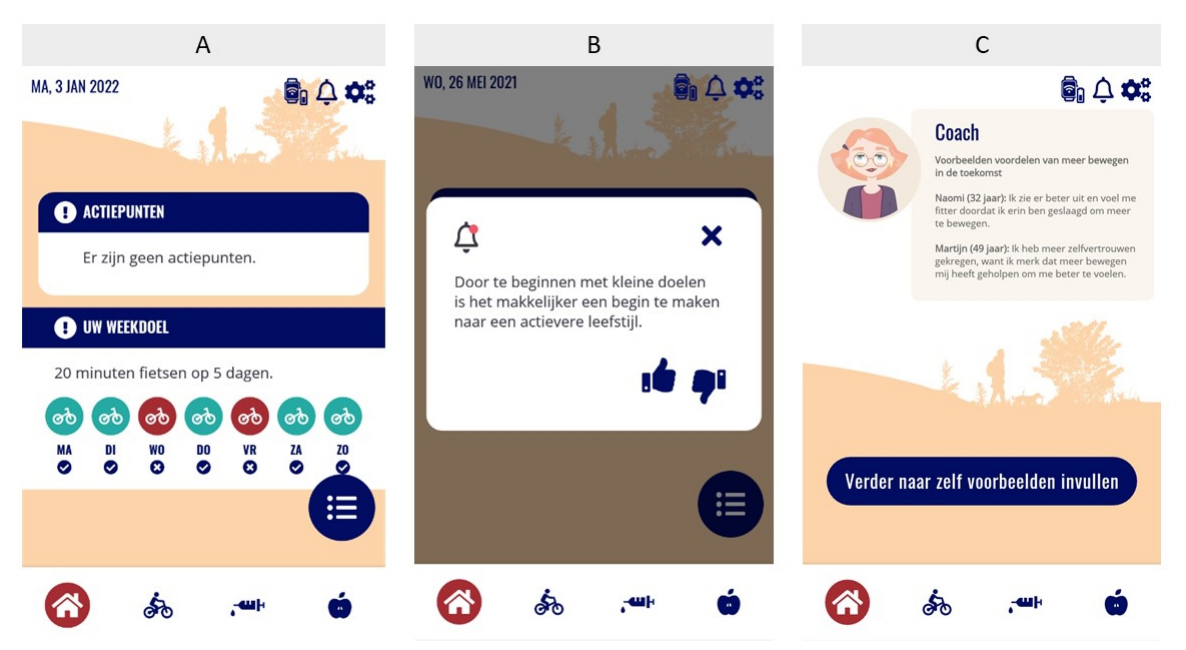
Screenshots from the Diameter app. A: home screen with overview of goal achievement; B: example of motivational message as push notification; C: example of start screen of an exercise.

Diameter is a Dutch app for people with T2D who aim to improve their glucose regulation through lifestyle changes. To date, the Diameter app has been used in research as a blended care intervention in secondary hospital care. All monitoring and coaching components of E-Supporter were integrated into Diameter app. Motivational messages were integrated as push notifications that could be closed by liking, disliking, or the closing button. Goal setting and weekly exercises that focused on barrier identification and problem-solving were integrated in the form of an interactive dialog with a conversational agent (Figure 4).

In addition to be coached via the E-Supporter content, individuals using the Diameter app could monitor physical activity (ie, with an activity tracker and manually), nutrition (ie, with an electronic food diary), and real-time glucose levels (ie, by using a Freestyle Libre 2 glucose sensor) [93]. The data collected with the app and sensors were fed back to health care providers so that these data could be used to conduct a more personal and patient-centered conversation based on objective data. Figure 4 shows some screenshots of the Diameter app.

##### Integration Within MiGuide

**Figure 5:**
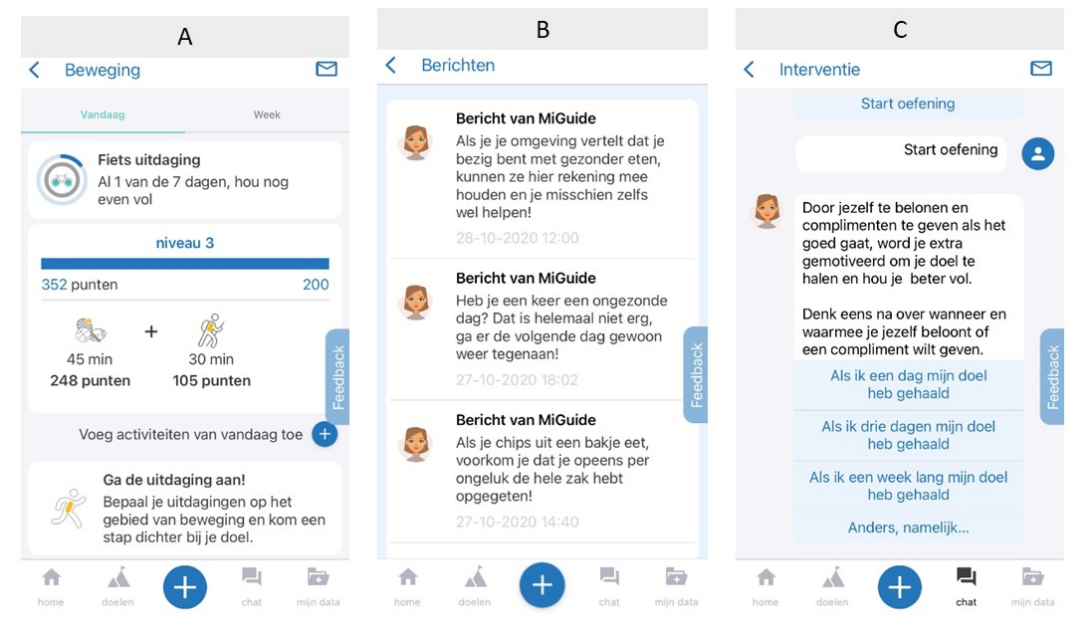
Screenshots from the MiGuide app. A: activity screen within MiGuide; B: examples of motivational messages within MiGuide; C: example of an exercise to increase motivation.

MiGuide is an app for people with T2D with the aim to improve lifestyle. The MiGuide app is available in both the Google Play Store and AppStore, is available in Dutch, and is offered in a blended care setting in primary care. Only the weekly psychological exercises aimed at barrier identification and problem-solving were integrated from E-Supporter 1.0 into the MiGuide app. MiGuide uses its own goal-setting options, self-monitoring tools, and short messages similar to E-Supporter. In the app, previously set goals, physical activity (ie, using an activity tracker and manually), nutrition (ie, using an electronic food diary), and glucose levels (ie, entering measured values manually) could be monitored. Coaching was offered through short messages developed by MiGuide and the psychological exercises from E-Supporter. The MiGuide app could be linked to different General Practitioner Information Systems (in Dutch: Huisarts Informatie Systeem). This allowed data to be exchanged between the MiGuide app and the Huisartsen Informatie Systeem. Patients could view their medical file via the app, and health care providers could gain insights into the measurements of their patients so that more personalized care could be offered. Figure 5 shows some screenshots of the MiGuide app.

### Phase 3: Review Phase

#### Participant Characteristics

In total, 9 participants (n=7, 78% male) were included in the usability study. Participants were on average 65.2 (SD 8.7) years old and obese (mean BMI 31.7, SD 3.29 kg/m^2^). On average, the participants had been diagnosed with T2D for 17 years. Diabetes-related complications were present in 67% (6/9) of participants. In total, 44% (4/9) of participants had used an app to track their physical activity or diet previously at the time of inclusion in the study.

#### Intervention Use

##### Self-monitoring Tools of the Diameter App

All (9/9, 100%) participants scanned the Freestyle Libre more than the requested 3 times per day with an average of 11 (SD 2.7) scans per day. Data loss occurred in 11% (1/9) of participants because the Freestyle Libre detached prematurely (after a week) from the upper arm; 11% (1/9) of participants experienced problems with synchronizing the Fitbit with the app. The participants without synchronization problems had an average of data loss of 24.8% (range 0%-66.4%). In addition, 67% (6/9) of participants logged activities in addition to using Fitbit. The food diary was completed by 89% (8/9) of participants for the requested 6 days.

##### E-Supporter Content

Of the 9 participants, 7 (78%) participants experienced problems receiving the 2 daily motivational messages; 1 (11%) participant received no messages at all, which could be explained by a human error made by the researcher. The other 6 participants received the messages very irregularly (eg, 1 instead of 2 messages per day or no messages at all for a day). The log data showed that the push notifications for the messages were sent from the app but that participants mentioned not to receive a notification on their phone. This issue was probably caused by phone settings or notification blockers (ie, push notifications marked as spam are killed by the system). Of the motivational messages of which participants did receive a push notification, 97.1% (298/307) were read. Participants liked 73.8% (220/298) and disliked 12.1% (36/298) of the content of the motivational messages. The content of the remaining messages was perceived as neutral (42/298, 14.1%). No major differences were found between participants. In total, 43% (17/40) of the psychological exercises were completed, with a broad variety in completion rates between participants (range 0%-100%). Furthermore, it was noted in the log data that missing data (eg, missing Fitbit data and missed EMAs) were included in the weekly feedback by stating that a goal was not achieved on the days when there were missing data.

#### Acceptability

##### Overview

Experiences with E-Supporter 1.0 integrated within the Diameter app were reduced to two major themes: (1) *Content of the E-Supporter 1.0* and (2) *Way of delivery via the Diameter*. The theme *Content of the E-Supporter 1.0* included perspectives on the content of E-Supporter 1.0 (eg, opinions on information and advice provided). The theme *Way of delivery via the Diameter* was related to the way the E-Supporter functionalities were integrated in the Diameter app and experiences with the additional self-monitoring tools that were offered with the Diameter app.

##### Content of E-Supporter 1.0

Participants experienced the goal-setting functionalities as motivating because it gave them a concrete purpose to work on. No areas for improvement were identified for the goal-setting functions. Motivational messages were mostly positively rated. Participants called the coaching messages fun, motivating, and informative and believed that it also contributed to lifestyle improvements. The content of the intervention options matched their preferences regarding the type of physical activities and diet well, but the participants felt that the content could be further tailored. Preferably, the participants would also like to receive real-time feedback on their actual behavior (eg, feedback whether a certain amount of physical activity is sufficient). Opinions were divided about the psychological exercises (ie, conversational agent). Some participants saw the added value of these exercises and thought it made them think about how to achieve their goals as participant 6 mentioned the following:

I like the online coach. Then you become more aware of your own behavior. I mainly use it to think about how I want to take more steps and the coach does make you think about that.

Male, 52 years

However, other participants found the purpose of the weekly exercises unclear which demotivated them to complete the exercises. Participant 3 explained why the conversational agent was not of added value for her:

I think that is a bit of a nagging of “the coach wants to talk to you” [push notification to complete the psychological exercise]. I do not need that. Then I have that message again and then I think quickly write in some answers and then we are done with it. I don’t see the added value in that, because I can also think for myself why I will or will not achieve my goal.

Female, 54 years

##### Way of Delivery via the Diameter App

The participants considered the self-monitoring functionalities of the Diameter app to be valuable because they provided new insights into their own lifestyle. For example, several participants indicated that through self-reporting and tracking physical activities they learned that they were not as active as they thought. In addition, all the participants indicated that the self-monitoring functionalities gave them insight into the effects of lifestyle on glucose levels as participant 2 said the following:

The Diameter provides insight, for example which activities you have undertaken, which food you have eaten and what impact that has had on glucose values. That is extremely useful.

Male, 66 years

These kinds of insights convinced the participants that an improvement in lifestyle can lead to improved glucose regulation. For several reasons, participants expected that the Diameter app could be a valuable addition to regular care. First, participants stated that the Diameter app could support the transition to a healthy lifestyle by complementing the information and advice of health care professionals during the consultations in the hospital. Second, participants thought that it could be beneficial if health care providers could also access the collected data. Participants believed that insight into these data could allow health care professionals to provide more personalized lifestyle advice. Furthermore, some participants noted that sharing data with health care providers could also lead to more proactive care as participant 6 outlined the following:

If healthcare providers have insight into my data, they can act much more proactively. This means that I do not have a consultation with the doctor every few months, but that consultations will be planned, when necessary, for example if my blood sugars are poorly regulated. But also, regular consultations will be omitted if everything goes well.

Male, 52 years

Participants indicated that they experienced a high degree of user-friendliness because each component (ie, physical activity, nutrition, and glucose levels) had its own tab and there were a limited number of buttons. The biggest point of criticism regarding ease of use was filling in the food diary. It was difficult to find certain foods or these were not available at all in the food diary. This was particularly experienced by participants who often eat dishes from foreign cuisine. As a result, participants had to look for alternative foods that are similar. This took a lot of time, and participants felt that this gave a distorted picture of their diet. Other frequently mentioned disadvantages were related to the way E-Supporter 1.0 was integrated within the Diameter app. It was not possible to read the motivational messages again once they had closed the message as participant 8 echoed the following:

Sometimes I wanted to read the information from the messages again later or I wanted to look up the hyperlink to a website again. That was not possible now. That is a pity because then I cannot do anything with it anymore.

Female, 73 years

Regarding the weekly psychological exercises, participants found it inconvenient that the exercises came at a fixed day and time in the week and could not be postponed to another moment. In addition, the exercises popped up automatically on their screen, making it impossible to perform another action within the app (eg, filling in the food diary) without completing the exercise. Participants would like to be able to choose at what time they perform the exercises so that they could also take the time to go through it carefully.

All detected bugs, inconveniences, and so on were listed and fixed by the app developers accordingly whenever possible.

## Discussion

### Principal Findings

This study describes the development of E-Supporter 1.0, a lifestyle monitoring and coaching intervention, using a systematic and participatory 3-phase design approach. The aim of E-Supporter 1.0 was to encourage people with T2D to be physically active and to follow national dietary guidelines. The HAPA model and theories explaining behavioral maintenance were used to select determinants of behavior and identified BCTs that were presumed to affect the targeted determinants. Thereafter, the intervention was developed by (1) selecting targets to tailor the intervention, (2) operationalizing decision rules to provide the right type of intervention option to an individual, and (3) creating intervention options to influence health behavior.

Regarding intervention development, we ensured a systematic and participatory design approach. The use of program-planning models provided detailed guidance on how to develop E-Supporter 1.0. This approach increased transparency in the design process by providing a comprehensive description of the intervention rationale and development of intervention components. This contributes to a better interpretation of results and the replicability of the intervention [59] and may serve as inspiration for other researchers [154]. Furthermore, health care professionals and people with T2D participated at several moments in the development process. Several studies [155-158] noted the importance of involving end users and other stakeholders in activities related to the development, implementation, and evaluation of eHealth interventions. Development “with” end users or other stakeholders increases the chances of successful adoption of and engagement with eHealth interventions, which in turn increases the likelihood of achieving desired effects. In our study, the involvement of health care professionals and people with T2D provided useful input regarding the requirements, development, and improvement of the intervention. For example, because of the focus groups with health care professionals, the content, readability, and comprehensibility of the motivational messages were improved so that these may have a better fit with the target group. The overall development approach, using program-planning models and participatory design, can facilitate future adjustments and development of the intervention.

So far, most of the eHealth interventions to promote health behaviors have shown positive short-term effects [40-46]. E-Supporter goes beyond many existing eHealth interventions by integrating 3 evidence-based elements that could increase intervention effectiveness. First, theory-driven methods were used as the fundament for the intervention [46,60-63]. Most eHealth interventions mainly focus on intention forming [67,159]. However, individuals often do not act in accordance with their intentions (ie, intention-behavior gap) [160], and behavior often cannot be maintained in the long term [112]. Therefore, our intervention also focuses on behavior initiation and maintenance in addition to intention forming [111,112,145,161] by covering determinants (eg, coping planning) and BCTs that target postintentional phases [65,117]. Second, dynamic tailoring was applied to increase the probability of adherence to and effectiveness of the intervention [69,70]. Dynamically tailored interventions provide support that better meet user needs than static tailored interventions [39]. Therefore, dynamic tailoring may increase feelings of personal relevance and responsiveness to the intervention option. Third, our intervention content was integrated into app-based interventions that are used in a blended care setting in primary and secondary care. There is a growing body of literature that recognizes that blended interventions are more likely to be used and effective than stand-alone interventions [36,75-77,79-81]. By combining aforementioned elements, we expect that our intervention could positively contribute to sustainable health behavior change, although this still needs to be researched.

In addition to individual factors, behavior is largely influenced by the (social) living environment [162-164]. Both the physical (eg, food supply and availability of sports and recreational facilities) and social environment (ie, the behavior of people in the environment) can contribute to the formation of certain barriers to a healthy lifestyle [24,162]. E-Supporter offers tools and techniques on how to deal with these (social) environmental barriers, for example, by indicating how individuals can organize social support in daily life or can learn to deal with social influences on lifestyle choices. By using eHealth, people’s attitudes toward unhealthy lifestyle behaviors can change and people can learn to deal with barriers in the social environment through coping strategies. However, in many cases, the (social) living environment will not change substantially (eg, the presence of sports facilities and social influences). A (social) living environment that tempts unhealthy behavior therefore remains an important barrier to successful adoption and maintenance of a healthy lifestyle.

A feature that distinguishes E-Supporter from other eHealth interventions is that E-Supporter content can be integrated in different eHealth interventions and settings. Although this version aimed to improve physical activity and diet in people with T2D, we expect that the content can be used to promote other lifestyle behaviors in people with other chronic diseases with simple adjustments owing to about 80% of the content consisting of nondisease-specific health information. Moreover, often the same behavioral determinants influence the behavior change process (eg, self-efficacy).

### Strengths and Limitations

The strength of this study is that our intervention combines several potentially effective elements for eHealth interventions, including the application of behavior change theory and dynamic tailoring, the deployment of the intervention in a blended care setting, and early end user involvement in both intervention development and evaluation. Another strong point of E-Supporter is that the content can be built into different apps so that it can be used in different contexts and possibly also on a larger scale.

Some limitations are worth noting at this stage of the research. The weekly feedback on goal achievement and whether to offer psychological exercises relies highly on input from the user. Lifestyle goals other than step goals were actively monitored through daily EMAs, which rely on an individual’s daily response. Therefore, work is being done on this issue by making more use of passive assessment tools that require minimal user input (eg, using activity trackers to track cycling goals). In addition, if individuals provided insufficient input, the Diameter app based the tailored feedback on incomplete information. This led to individuals receiving inappropriate feedback that they had not sufficiently achieved their goals, which can be demotivating to use the intervention. The Diameter app was technically adjusted so that no feedback will be given if there is insufficient user input to provide valid feedback.

We had little influence over the design and user interface of the Diameter app into which the E-Supporter content was integrated. The design and interface of the intervention can influence the user experience and use of the app both positively and negatively [165,166]. Our usability test showed that the interface of certain E-Supporter elements in the Diameter app negatively influenced the experience with E-Supporter (eg, automatic pop-up of the psychological exercises). However, it is not clear to what extent a different design and interface (eg, through integration in another app such as MiGuide) will lead to different findings regarding the acceptability of E-Supporter. The content of E-Supporter remains unchanged, but some aspects of the intervention (eg, attractiveness or ease of use) may be experienced differently.

During the usability study, we encountered some challenges in the technical integration of E-Supporter 1.0 into the Diameter app (eg, receiving motivational messages irregularly). These challenges led to the intervention not being delivered as intended, which negatively influenced the results regarding use and acceptability of E-Supporter. To improve the integration, we recorded all detected technical problems and discussed them with the app developer to solve them. Lessons learned from this usability test will be used to make recommendations regarding the integration of E-Supporter content in apps to promote a positive user experience. For example, it is necessary to test what influence missing data has on the feedback initiated by the app (eg, as described in the first limitation).

### Future Research

We have planned several follow-up activities to further improve E-Supporter. First, the intervention content and intervention period will be expanded so that E-Supporter 1.0 can better facilitate behavioral maintenance. Literature states that behavior maintenance is reached when an individual can maintain the desired behavior for at least 6 months [167]. To achieve behavior maintenance, it is challenging to offer digital coaching over a long period without losing adherence to the intervention, given the high attrition rates in eHealth use over time [168-170].

Second, researching the use of BCTs in other delivery modes (eg, videos and voice messages) than textual coaching is suggested. The content of the intervention options can remain unchanged but will be offered via a different delivery mode. Other delivery modes can make the intervention options more attractive to increase acceptance [171] and more comprehensible to people with low (health) literacy.

Third, we intend to tailor our intervention more dynamically by applying data science techniques because higher degrees of tailoring can contribute to improved user engagement and effectiveness [31,63,172,173]; for instance, by tailoring the intervention content to additional determinants of behavior, individual characteristics (eg, health literacy), current behaviors, or predicted high-risk situations (eg, as in just in-time adaptive interventions) [39]. To optimize tailoring strategies in future, we can examine the effects of intervention options on proximal outcomes (ie, short-term goals) per individual and whether these effects vary with time or circumstances using study designs such as microrandomized trials [39,174]. In microrandomized trials, individuals are randomized hundreds of times over the course of the study by being randomly assigned to an intervention option at a decision point (ie, time points when an intervention decision must be made). We are already working in a multidisciplinary research team involving, among others, behavioral health experts, health care professionals, and computer science specialists, which is required to develop highly tailored behavior change interventions [31,39,175].

Fourth, our intervention can be improved by aligning intervention content of the ABCC tool and E-Supporter (Figure 1). E-Supporter can be expanded with content for additional target groups (eg, people with chronic obstructive pulmonary disease or chronic heart failure) and lifestyle behaviors (eg, smoking) that are part of the ABCC tool. Moreover, data exchange between the ABCC tool and E-Supporter should be made possible so that the eManager can be used as an integrated blended care intervention.

To obtain more information about the intervention use and acceptability of our intervention, we plan to evaluate the E-Supporter content in other apps, such as MiGuide [91]. This option allows us to investigate whether another design, interface, and functionality will result in different findings on some aspects (eg, attractiveness or ease of use) regarding the experience with the E-Supporter. At the end of 2022, the effectiveness of E-Supporter 1.0 will be explored in a blended care setting through a single-arm longitudinal study with 6-month follow-up. Finally, new versions of E-Supporter will have to be iteratively evaluated regarding user engagement and cost-effectiveness in the long run [31].

### Conclusions

This paper describes the systematic and participatory development of a theory-based, dynamically tailored lifestyle coaching intervention to support physical activity and a healthy diet in people with T2D. Program-planning models and behavior change theory were used complementarily during the development of the intervention. The intervention was evaluated in a small usability study which provided insights into intervention use and acceptability. Future work should focus on improving the degree of tailoring and evaluating its effects on acceptability, use, and cost-effectiveness.

## Appendix

Multimedia Appendix 1 Methods E-Supporter health expert review.

Multimedia Appendix 2 Methods E-Supporter usability study.

Multimedia Appendix 3 E-Supporter determinants and behavior change techniques (BCTs) including examples.

Multimedia Appendix 4 E-Supporter psychological exercises.

## Abbreviations

ABCC: Assessment of Burden of Chronic Conditions
BCT: behavior change technique
EMA: ecological momentary assessment
HAPA: health action process approach
QoL: quality of life
T2D: type 2 diabetes
