# Supplementary material for: A Digital Lifestyle Coach (E-Supporter 1.0) to Support People With Type 2 Diabetes: Participatory Development Study
Source: JMIR Hum Factors. 2023 Jan 12;10:e40017. doi: 10.2196/40017 (PMC9947918; doi:10.2196/40017)
Supplement: Multimedia Appendix 1 [file humanfactors_v10i1e40017_app1.pdf]

Multimedia Appendix 1. Methods E-Supporter Health Expert Review

The text messages of the E-Supporter were reviewed by a panel of HealthCare Professionals (HCPs) during two focus group discussions to optimize the content of the E-Supporter before it was pilot tested by a group of patients. We recruited a heterogeneous panel of HCPs, including internists, nurse practitioners and diabetes nurses who performed two assignments to review the intervention content.

Exercise: "Good" or "Need to adapt"

Within the first assignment, HCPs had to assign the messages into the two groups, "approved" message or "need to adapt" message, based on content and readability of the message (see Figure 1). The participants were instructed to assign a message to "approved message" group if they considered the content of the message as substantively correct and understandable for a person with T2D. Otherwise, the message was categorized in the "need to adapt" group. The latter messages were discussed plenary to either adapt the content into a "approved message" or to exclude the message from the database.

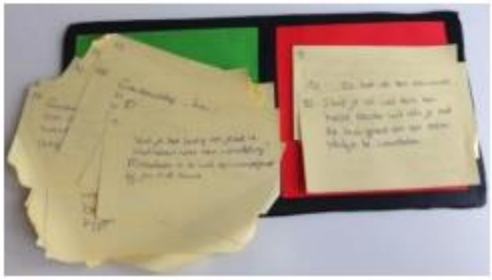

Figure S1. The board with text messages as a result of the *"Good" or "Need to adapt"* exercise
