# Supplementary material for: A Digital Lifestyle Coach (E-Supporter 1.0) to Support People With Type 2 Diabetes: Participatory Development Study
Source: JMIR Hum Factors. 2023 Jan 12;10:e40017. doi: 10.2196/40017 (PMC9947918; doi:10.2196/40017)
Supplement: Multimedia Appendix 2 [file humanfactors_v10i1e40017_app2.pdf]

Multimedia Appendix 2. Methods E-Supporter Usability Study

Since the E-Supporter's initial design and application will be used to support people with diabetes, we evaluated the E-Supporter 1.0 in an usability study among people with Type 2 Diabetes (T2D). The E-Supporter content was integrated in a mobile app to support a healthy lifestyle in people with T2D, called the Diameter.

1. Study aims

The aim of this study was twofold: (1) to gain insight into intervention usage and acceptability of the E-Supporter 1.0 integrated in the Diameter and (2) to identify technical issues in the integration of the E-Supporter 1.0 within the Diameter app.

2. Study design

The usability study was a monocentre, mixed-methods cross-sectional study to examine intervention usage, acceptability, and technical issues of the E-Supporter 1.0 integrated in the Diameter.

3. Intervention

The participants used the Diameter app, with the E-Supporter content, for a period of 5 weeks at home. As part of the Diameter self-monitoring functionalities, participants monitored their physical activities (with a Fitbit activity tracker [1]), nutrition (digital food dairy within the Diameter app) and glucose levels (with Freestyle Libre 2 sensors, a continuous glucose monitoring sensor in the interstitial fluid of the upper arm [2, 3]). The Freestyle Libre 2 sensor was one of the self-monitoring tools of the Diameter app to provide people with T2D with continuous insight in glucose values. Regarding the E-Supporter components, participants received digital personalized coaching with a maximum of 2 motivational messages per day and 1 psychological exercise per week about goal setting and achievement. Afterwards, participants received personalized feedback on the measured glucose values (i.e., overview of measured glycemic parameters) and lifestyle (i.e., overview of daily step count and dietary intake) by one of the researchers or a healthcare professional.

4. Study population

The aim was to include 8 to 10 patients with T2D in the pilot study. We assumed that a group of 8 to 10 participants should be sufficient to gain insight into intervention usage and acceptability of the E-Supporter integrated in the Diameter. Patients were recruited in ZGT hospital, Almelo, The Netherlands. The patients' population consisted of 9 T2D patients (18+, male and female) visiting the outpatient clinic for T2D at ZGT hospital. We included patients who use blood glucose lowering medication independent of their gender and socio-economic status. Based on a baseline measurement of 450 patients with T2D treated in ZGT, the patients are on average 63 years old and 58% are male. On average, the patients have T2D for 11 years and a BMI of 32.9 kg/m^2 ± 6.2.

4.1 Inclusion criteria

To be eligible to participate in this study, a patient must:

- have been diagnosed with T2D;
- have an Android smartphone (version 5.0 or higher);
- be 18 years or older;

- have the intellectual capacity to understand what participation in the study entails;
- provide written informed consent.

4.2 Exclusion criteria

A potential subject will be excluded from this study if the patient:

- is dependent on kidney dialysis;
- has been diagnosed with serious illnesses or psychological disorders that make participation in the study impossible;
- is familiar with drug abuse (which will be assessed on the basis of history);
- insufficient command of the Dutch language.

5. Data collection

5.1 Intervention usage

Intervention usage was explored to gain more insight whether the intervention was used as intended. Intervention usage was assessed using log data of the several Diameter components (ie, Freestyle Libre 2 sensor, Fitbit activity tracker, food diary) and E-Supporter components (ie, motivational messages, feedback, psychological exercises).

5.2 Acceptability

Acceptability of the E-Supporter integrated into the Diameter was assessed by means of open-ended interviews. A semi-structured interview guide including topics derived from the Unified Theory of Acceptance and Use of Technology 2 (UTAUT2)-model [4] was used to capture participants' experiences with the E-Supporter integrated into the Diameter. Table S1 provides an overview of the topics included in the interview guide. Participants were asked a series of open-ended questions about the topics. An example of an open-ended question was: "What did you think of the Diameter's ease of use?". The interview guide included various follow-up questions to help elicit relevant experiences to answer the research question.

Table S1. Main topics of the interview guide

Number	Topics	UTAUT determinant
**1**	General impression of the Diameter	-
**3**	Ease of use of the Diameter (including technical issues, interface design)	Effort expectancy
**4**	Perceived usefulness of the Diameter	Performance expectancy
**5**	Perceived usefulness of the E-Supporter content	Performance expectancy
**6**	Appreciation and perceived enjoyment of E-Supporter content	Hedonic motivation
**7**	Technical infrastructure to use the Diameter	Facilitating conditions
**8**	The Diameter in the healthcare process	-

5.3 Background characteristics

Socio-demographic and medical characteristics were collected during the visit to the outpatient clinic. Characteristics of interests were: age [year], gender [m\v], Body Mass Index (BMI) [mean, SD], years of being diagnosed with T2D [years], and presence of diabetes-related complications [yes/no].

6. Study procedure

Table S2 describes the study procedure for the usability study in ZGT hospital. The study started with an extension of a regular visit to the ZGT outpatient clinic. Participant first received information about the study procedure and signed the informed consent. Thereafter, participant received an explanation about the Diameter, Fitbit and Freestyle Libre and, if necessary, received support in installing the Diameter and connecting with the Fitbit. The Diameter was in the home setting for a period of 5 weeks. Participants were asked to use the Diameter as intended by the research team. For this, participants were asked to scan the Freestyle Libre sensor at least 3 times a day (to prevent data loss), to wear the Fitbit activity tracker every day, and to fill in the food diary for at least 6 days. In addition, participants were requested to read the motivational messages and to make the weekly psychological exercises when offered. In week 6, participants received personalized feedback on the collected data regarding glucose values, physical activity and nutrition via a healthcare professional. Subsequently, the semi-structured interview was conducted. Participants first got an introduction into the aim and nature of the interview followed by the interview questions based on the interview guide. All interviews were audio-recorded. The nine interviews were conducted from March to July 2021 and took place either face-to-face at ZGT hospital or via video calling or by phone. The interviews took between 20 and 40 minutes, excluding the introduction.

Table S2. Study procedures for the usability study

Moment	Location	Time	Procedures
Start of the study	Outpatient clinic (extension of regular visit)	Day 1	• Explanation of the study.<br>• Singing of the informed consent form<br>• Instruction manual of the Diameter.<br>• Freestyle Libre sensor scan and placement instructions.<br>• Freestyle Libre Pro sensor will be placed on the upper arm.<br>• Explanation of the use of the Fitbit and connection with the Diameter.<br>• Explanation about intended use.
Use of the Diameter	Patient's home	Week 1-5	• Patients wore the Fitbit wristband for 5 weeks.<br>• Patients wore the Freestyle Libre sensor for 2 weeks.<br>• Patients used the Diameter to enter their food and drink intake for at least 6 days (time, type and quantity of food intake).<br>• Patients received a maximum of 2 motivational messages a day via the Diameter about physical activity and/or nutrition.<br>• Patients received a weekly psychological exercise containing feedback and an exercise that support people in achieving lifestyle goals.
Primary endpoint (T1)	Outpatient clinic/ (video) call	Week 6	• Feedback on collected data with regard to the patient's lifestyle parameters (e.g. glucose values, activity, nutrition).<br>• (Digitally) conducted open-ended interview to capture experiences with the Diameter and E-Supporter 1.0.

7. Data analysis

6.1 Intervention usage

Usage of the Diameter and E-Supporter components was reported by describing frequency and duration of the used functionalities by using log-data. In addition, we reported whether the intervention was used as intended.

6.2 Acceptability

Interviews were recorded in audio and transcribed non-verbatim. Inductive thematic analysis [5] was used to code the transcripts. First, the transcripts were read independently by 2 researchers to select relevant fragments that related to the research question. Various codes and sub codes were inductively created based on the selected fragments. Second, the codes were reviewed and split, combined, added or removed to optimize the codes. The coding of the transcripts were compared and in case of disagreement, both researchers discussed until agreement was reached. Third, all codes were examined for similarities and grouped inductively into general themes. Lastly, the themes found were revised and refined until it gave a good representation of the data.

8. Non-investigational products

Fitbit Inspire 2

The Fitbit is a wearable activity tracker worn on the wrist and measures the amount of steps per minute using 3D accelerometry [1]. They measure continuously the heart rate with its PurePulse LED lights. An algorithm developed by Fitbit measures the amount of heart beats per minute and the amount of steps per minute.

Freestyle Libre 2 glucose sensor

The Freestyle Libre glucose sensor, developed by Abbott, is a waterproof adhesive medical device [2]. The sensor has a small needle which measures glucose values in the interstitial fluid, between the skin and muscle layers, on the upper arm for a maximum duration of 2 weeks. Freestyle Libre 2 offers real-time minute-by-minute readings and stores readings every 15 minutes [3]. Data is stored on the sensor up to 8 hours and a reading device (reader kit)/application can extract and store up to 30 days of data. One sensor lasts up for 14 days. In addition, Freestyle Libre 2 includes optional, real-time glucose alarms that alert someone when someone is experiencing hyper- or hypoglycemia. No fingerstick calibration is required.

9. Ethical aspects

Ethical approval was obtained from the Medical research Ethics Committees United (MEC-U) Nieuwegein, the Netherlands (R20.121). Written consent was requested from each patient to participate in the study. Participants were informed in detail about how data were collected, processed and stored in the subject information sheet. Participants gave explicit consent for the use of their data by signing the informed consent. Data privacy was protected by offering anonymous pre-set accounts without personal data to prevent sharing personal data with commercial parties.

References

1. Fitbit Inc. Inspire 2. URL: https://www.fitbit.com/global/us/products/trackers/inspire2 [accessed 2022-02-16].
2. Abbott Inc. Freestyle Libre. 2022. URL: https://www.freestyle.abbott/nl-nl/home.html [accessed 2021-01-18].
3. Abbott Inc. Freestyle Libre 2. 2022. URL: https://www.freestyle.abbott/nl-nl/producten/FreeStyle-Libre2.html [accessed 2022-04-13].
4. Venkatesh V, Thong YL, Xu X. Consumer acceptance and use of information technology: extending the unified theory of acceptance and use of technology. MIS Q 2012 Mar;36:157-178. [doi: 10.2307/41410412]
5. Braun V, Clarke V. Using thematic analysis in psychology. Qual Res Psychol 2006 Jan;3:77-101. [doi: 10.1191/1478088706qp063oa]
