# Supplementary material for: A Digital Lifestyle Coach (E-Supporter 1.0) to Support People With Type 2 Diabetes: Participatory Development Study
Source: JMIR Hum Factors. 2023 Jan 12;10:e40017. doi: 10.2196/40017 (PMC9947918; doi:10.2196/40017)
Supplement: Multimedia Appendix 3 [file humanfactors_v10i1e40017_app3.pdf]

Multimedia Appendix 3. E-Supporter Determinants and BCTs Including Examples

Table S1. Determinants, BCTs and intervention features of the E-Supporter

	Determinants and BCTs	Content in E-Supporter	Intervention feature(s)	Example(s)
Key determinants	**Action control:** A self-regulatory strategy where the behavior is continuously evaluated with regard to a behavioral standard [1].			
	1.5 Review behavior goal [2-4]	Review of the behavior goal and modifying the goal in light of achievement	Goal setting options<br>Intervention option: feedback	"In the past week, your goal was to eat two pieces of fruit every day. You achieved your goal in 5 days. Do you want to adjust your current goal, set a new goal or preserve your current goal for the coming week?"
	2.2 Feedback on behavior [3-8]	Provide informative and evaluative feedback on performance of the behavior in in relation to the set lifestyle goal	Intervention option: feedback	"Hi *[name]*, let's take a look back at how it went last week. You achieved your goal in 6 days this week."
	2.3 Self-monitoring of behavior [3-5, 9, 10]	Provide a person with methods to monitor and record their behavior(s) in relation to the set lifestyle goal	Self-monitoring tools	• Track steps with activity tracker<br>• Track physical activity with digital activity diary<br>• Track nutrition with digital food diary
	**Self-efficacy:** One's subjective perception of his or her capability to perform in a given setting or to attain desired results [1, 11].			
	1.1 Goal setting (behavior) [12]	Set a weekly goal defined in terms of increasing physical activity or better adherence to dietary guidelines	Goal setting options	"Think about how many steps you would like to take per day. At the moment you take an average of 4,200 steps per day.<br><br>What do you think, how many steps would you like to take daily in the coming week?"
	1.2 Problem solving [12-14]	Prompt the analysis of factors that influence the behavior and select strategies to overcome barriers or increase facilitators (includes "Coping Planning")	Intervention option: motivational message	"Did you know that creating an if-then plan can help you resist temptation for unhealthy products? Determine for yourself the temptation (if…) and how you want to deal with it (then…)."
	1.4 Action planning [12, 14, 15]	Prompt planning the performance of a particular physical activity or eating a particular meal at a particular time at certain moments (includes "Implementation Intentions")	Intervention option: motivational message	"Good morning *[name]*, an action plan can help you to become more active. Think about what you are going to do (eg, I want to be more active). How are you going to do it (eg, I'm going to do something I like)? Which activity (eg, walking)? When (eg, after dinner)? How long (eg, 20 minutes)?
	2.3 Self-monitoring of behavior [12]	Provide a person with methods to monitor and record their behavior(s) in relation to the set lifestyle goal	Self-monitoring tools	• Track steps with activity tracker<br>• Track physical activity with digital activity diary<br>• Track nutrition with digital food diary
	3.1 Social support (unspecified) [12, 14, 15]	Advise on arranging social support from relatives, friends or colleagues for performance of physical activity or following a healthy diet	Intervention option: motivational message	"If you tell the people around you that you are working on healthier eating, they can take this into account and maybe even help you!"

	4.1 Instruction on how to perform the behavior [13, 14]	Advise on what physical activities and foods/nutrients to pursue and how	Intervention option: motivational message	"Good morning *[name]*, Good preparation can help to get enough exercise. For example, check the weather forecast in the morning and plan your walk when the chance of rain is low."
	11.2 Reduce negative emotions/ increase positive emotions [a] [12]	Advise on ways of reducing negative emotions or increasing positive emotions to facilitate performance of physical activity or a healthy diet	Intervention option: psychological exercise	By reflecting on nice things that you experience, you become happier. Think of 3 fun things that happened to you today or yesterday. *<enter field>*<br><br>You experienced 3 things that make you happier. If you want, you can make such a list at the end of every day for the next week. Hopefully this helps you to think positively.
	15.1 Verbal persuasion about capability [13]	Tell that the person can successfully perform physical activity or follow a healthy diet despite self-doubts	Intervention option: motivational message	"Good morning *[name]*, Did you not achieve your goal yesterday? That is part of wanting to live healthier. It doesn't go well every day. Don't let it stop you from reaching your goal. I'm sure you can do it today!"
	15.3 Focus on past success [13]	Advise to think about or list (reasons for) previous successes in performing the behavior	Intervention option: psychological exercise	The following exercise will help you reflect on how you have dealt with challenges before and how this can help you to *[healthy behavior]*. Have you ever managed to do something that was difficult? Enter your success experience below. *<enter field>*<br><br>Describe what you did that made it successful. This is what I did, so it still worked: *<enter field>*
**Initiation phase**	**Knowledge:** The state of being familiar with something or aware of its existence, usually resulting from experience or study [16].			
	4.1 Instruction on how to perform the behavior [3, 13]	Advise on what physical activities and foods/nutrients to pursue and how	Intervention option: motivational message	"If you're looking for reliable nutritional information, take a look at the website of "the Dutch Nutrition Center". They provide a lot of reliable information about healthy nutrition."
	5.1 Information about health consequences [3, 9, 13]	Provide information about (diabetes related) positive and negative health consequences of (not) performing physical activity or following healthy diet	Intervention option: motivational message	"Hi *[name]*, Did you know that physical activity reduces the risk of additional physical consequences of diabetes (complications)? Examples of these complications include damage to the feet, nerves, eyes, kidneys and heart and blood vessels."
	**Risk perception:** One's subjective judgments about the likelihood of negative occurrences such as injury, illness, disease, and death [1, 17].			
	5.1 Information about health consequences [12, 13]	Provide information about (diabetes related) negative health consequences of (not) performing physical activity or following healthy diet	Intervention option: motivational message	"You are more likely to develop cardiovascular disease if you sit a lot. It's good for you to stand up regularly. You burn 3 times more energy than when you are sitting."
	**Outcome expectancies:** Subjective estimates of how likely it is that a specific behavior will be followed by particular consequences (cognitive, emotional, and behavioral) [18].			
	5.1 Information about health consequences [9, 13]	Provide information about (diabetes related) positive and negative health consequences of (not) performing physical activity or following healthy diet	Intervention option: motivational message	"Hi *[name]*, we know from scientific research that you can reduce the risk of some cancers by eating healthy and varied. For more information, visit the Nutrition Center website!"

	5.6 Information about emotional consequences [3, 9, 13]	Provide information about emotional consequences of performing physical activity and following a healthy diet	Intervention option: motivational message	"Hi *[name]*, Regular physical activity can help you to get more energy. This in turn has positive effects on your mood, concentration and mental stress."
	9.2 Pros and cons [12, 13]	Advise for identification and comparison reasons for wanting and not wanting to change lifestyle	Intervention option: motivational message	"Hello *[name]*, List the advantages and disadvantages of more physical activity. Do the advantages outweigh the disadvantages for you?"
	9.3 Comparative imagining of future outcomes [13]	Advise the imagining and comparing of future outcomes of changed versus unchanged lifestyle behavior	Intervention option: motivational message	"Hello *[name]*, first imagine how your diabetes will be regulated in a year's time if you start being physically active from today. Then imagine how your diabetes will be regulated in a year's time if you decide not to adjust your activity pattern. What are your thoughts on this?
	**Attitude:** The general evaluations of the behavior on a scale ranging from negative to positive [19].			
	5.1 Information about health consequences [3, 13]	Provide information about (diabetes related) positive and negative health consequences of (not) performing physical activity or following healthy diet	Intervention option: motivational message	"According to TNO (research center), 2 out of 5 participants in a lifestyle program will eventually be able to live without medication."
	9.1 Credible source [13]	Provide written information from a credible source in favor of physical activity or a healthy diet	Intervention option: motivational message	"The World Health Organization (WHO) advises people to stay active on a daily basis in order to gain and maintain more control over their own health."
	9.2 Pros and cons [12, 13]	Advise for identification and comparison reasons for wanting and not wanting to change lifestyle	Intervention option: motivational message	"Do you sometimes feel that it is not worth changing your diet? Then write down all the benefits and drawbacks. Hopefully you will see that the benefits outweigh the drawbacks!"
	13.2 Framing/reframing [12, 13]	Suggest to adopt a (new) perspective on physical activity or a healthy diet (e.g. its purpose) in order to change cognitions or emotions about performing these behaviors (includes "Cognitive Structuring")	Intervention option: motivational message	"See being physically active as an opportunity to relax mentally rather than an obligation to be physically active."
	**Social support:** The provision of assistance or comfort to others, typically to help them cope with biological, psychological, and social stressors [20].			
	3.1 Social support (unspecified) [3, 9, 12, 13]	Advise on arranging social support from relatives, friends or colleagues for performance of physical activity or following a healthy diet	Intervention option: psychological exercise	"It helps to tell others that you have *[healthy behavior]*. This prevents you from ending up in situations that are tempting and therefore you do not achieve your goal. Practicing this in advance makes it easier 'in real life'.<br><br>Think about how you would like to do that.<br>Would you like to see an example of how to tell this? Then click on *[example]*."
	3.2 Social support (practical) [4, 9, 12, 13]	Advise on arranging practical social support from relatives and friends	Intervention option: motivational message	"Hi *[name]*, Do you often have insufficient time to put a healthy meal on the table? Consult with your housemates whether they can take

		for performance of physical activity or following a healthy diet		over other household tasks from you so that you have the time to cook healthy!"
**Action phase**	**Action planning:** A strategic method to plan the performance of the behavior (must include at least one of context, frequency, duration and intensity) [1, 19].			
	1.2 Problem-solving [3, 12, 21]	Prompt to identify factors influencing physical activity/healthy diet and to select strategies that supports overcoming barriers or increasing facilitators	Intervention option: psychological exercise	“Good preparation increases the chance that you will succeed in achieving your goal. Think about what situations are for you that tempt you into *[unhealthy behavior]*. And then look for solutions how to resist these tempting moments.”
	1.4 Action planning [4, 9, 12]	Prompt planning the performance of a particular physical activity or eating a particular meal at a particular time at certain moments (includes “Implementation Intentions")	Intervention option: psychological exercise	“By thinking about how you can manage to *[healthy behavior]*, you are more likely to actually succeed.<br><br>How would you manage to *[healthy behavior]*? Write down any ideas that come to your mind. Also the crazy, stupid and unfeasible ideas. They can give you a good and feasible idea.”
	7.1 Prompts/cues [9, 22]	Advising on arranging environmental stimulants with the aim of prompting and cueing physical activity/healthy diet	Intervention option: motivational message	“Hi *[name]*, write down your goal and put it in a visible place. This makes it concrete and ensures that you are often reminded of it!”
	**Coping planning:** Making specific plans to overcome anticipated barriers that may hinder individuals from enacting their intentions [1, 23].			
	1.2 Problem solving [3, 12]	Prompt the analysis of factors that influence the behavior and select strategies to overcome barriers or increase facilitators (includes “Coping Planning”)	Intervention option: motivational message	“Do you notice that bad weather stops you from doing physical activities outside? Then consider which activities you can do with bad weather. For example, household chores also count as physical activity.”
	1.4 Action planning [4, 12]	Prompt planning the performance of a particular physical activity or eating a particular meal at a particular time at certain moments (includes “Implementation Intentions")	Intervention option: motivational message	“You should have made an action plan to achieve your goal. Do you notice that your action plan is not working properly? Then see how you can adjust your action plan so that it has a better chance of success!”
**Maintenance phase**	**Mood management:** The act of trying to change or extend one's emotional attitude or disposition [24].			
	11.2 Reduce negative emotions [a] [13, 25, 26]	Advise on ways of reducing negative emotions or increasing positive emotions to facilitate performance of physical activity or a healthy diet	Intervention option: psychological exercise	Sometimes you are more concerned with things that are not going well than with things that are going well. But you feel happier when you think about the good things. Time to do something with that! Think about what makes you happy. It is best to compliment yourself for who you are or what you can do. Now write something nice to yourself: *<enter field>*<br>You wrote the following to yourself:<br>*<repeat answer>*<br><br>Pretty fun to read right?! Does this help you to dwell on good things? Then you can do that more often.

**Habits:** A well-learned behavior or automatic sequence of behaviors that is relatively situation specific and over time has become motorically reflexive and independent of motivational or cognitive influence—that is, it is performed with little or no conscious intent [27].			
1.2 Problem solving [3, 12]	Prompt the analysis of factors that influence the behavior and select strategies to overcome barriers or increase facilitators (includes "Coping Planning")		"Hi *[name]*, You've been working on getting more exercise for a while. Do you find it difficult for you to make a habit of this? Try to figure out why that is so difficult. What can help you make more exercise a habit?"
1.4 Action planning [12]	Prompt planning the performance of a particular physical activity or eating a particular meal at a particular time at certain moments (includes "Implementation Intentions")	Intervention option: motivational message	"Hi *[name]*, Have you been doing a "food swap" successfully for several weeks? Then you are probably ready for the next step! Come up with a clear plan. What are you going to do? How? When? And what do you do to resist the temptation for unhealthy food?"
7.1 Prompts/cues [4, 22]	Advising on arranging environmental stimulants with the aim of prompting and cueing physical activity/healthy diet	Intervention option: motivational message	"Do you ever set a new goal? For each new goal, try to find an environmental stimulus with the aim of encouraging healthy choices. Think, for example, of putting your sports shoes in plain sight or sticking a note on the fridge."
8.3 Habit formation [4, 12]	Prompt rehearsal and repetition of physical activities or nutritional habits in the same context repeatedly so that the context elicits the behavior	Intervention option: motivational message	"Have a piece of fruit at the same time or moment every day, for example during coffee break. You will find that it becomes a habit over time!"
**Satisfaction:** A summary assessment of the value of the experiences afforded by the new pattern of behavior and indicates that the initial decision to change the behavior was correct and worth sustaining [4].			
2.4 Self-monitoring of outcome(s) of behavior [4, 28]	Advise on using methods for monitoring the outcome(s) of their behavior	Intervention option: motivational message	"To maintain a healthy lifestyle, it can help to keep track of what you have already achieved. Keep track of for yourself, for example in a diary, whether a healthier diet results in weight loss. Write down your weight every time you weigh yourself!"
**Social influences:** Any change in an individual's thoughts, feelings, or behaviors caused by other people, who may be actually present or whose presence is imagined, expected, or only implied [29].			
3.1 Social support (unspecified) [12, 13]	Advise on arranging practical social support from relatives and friends for performance of physical activity or following a healthy diet	Intervention option: psychological exercise	"Do you ever struggle to maintain your healthy lifestyle? If you're having a hard time, ask a housemate or friend to encourage you."
3.2 Social support (practical) [12, 13]	Advise on arranging practical social support from relatives and friends for performance of physical activity or following a healthy diet	Intervention option: motivational message	"Maintaining a healthy lifestyle is difficult. It can help to have a regular buddy to exercise with. This makes you less inclined to stop after a few times."
6.3 Information about other's approval [12, 13]	Provide information about what other people think about physical or a healthy diet.	Intervention option: motivational message	"More and more people with diabetes find it important to work on a better lifestyle. Hopefully that is a good motivation to maintain your healthy way of life!"

[a] The additional technique 'Increase positive emotions', that will be included in BCT Taxonomy v2, was already used in this intervention.

References

1. Schwarzer R. Health action process approach (HAPA) as a theoretical framework to understand behavior change. Actual Psicol 2016 Dec 05;30:119-130. [doi: 10.15517/ap.v30i121.23458]
2. Avery L, Flynn D, van Wersch A, Sniehotta FF, Trenell MI. Changing physical activity behavior in type 2 diabetes: a systematic review and meta-analysis of behavioral interventions. Diabetes Care 2012 Dec;35:2681-2689 [FREE Full text] [doi: 10.2337/dc11-2452] [Medline: 23173137]
3. Dusseldorp E, van Genugten L, van Buuren S, Verheijden MW, van Empelen P. Combinations of techniques that effectively change health behavior: evidence from Meta-CART analysis. Health Psychol 2014 Dec;33:1530-1540. [doi:10.1037/hea0000018] [Medline: 24274802]
4. Rothman AJ, Sheeran P, Wood W. Reflective and automatic processes in the initiation and maintenance of dietary change. Ann Behav Med 2009 Dec;38 Suppl 1:S4-17. [doi: 10.1007/s12160-009-9118-3] [Medline: 19787308]
5. Sniehotta FF, Nagy G, Scholz U, Schwarzer R. The role of action control in implementing intentions during the first weeks of behaviour change. Br J Soc Psychol 2006 Mar;45:87-106. [doi: 10.1348/014466605X62460] [Medline: 16573874]
6. Michie S, Abraham C, Whittington C, McAteer J, Gupta S. Effective techniques in healthy eating and physical activity interventions: a meta-regression. Health Psychol 2009 Nov;28:690-701. [doi: 10.1037/a0016136] [Medline: 19916637]
7. Samdal GB, Eide GE, Barth T, Williams G, Meland E. Effective behaviour change techniques for physical activity and healthy eating in overweight and obese adults; systematic review and meta-regression analyses. Int J Behav Nutr Phys Act 2017 Mar 28;14:42 [FREE Full text] [doi: 10.1186/s12966-017-0494-y] [Medline: 28351367]
8. Van Rhoon L, Byrne M, Morrissey E, Murphy J, McSharry J. A systematic review of the behaviour change techniques and digital features in technology-driven type 2 diabetes prevention interventions. Digit Health 2020 Mar 24;6:2055207620914427 [FREE Full text] [doi: 10.1177/2055207620914427] [Medline: 32269830]
9. Abraham C, Michie S. A taxonomy of behavior change techniques used in interventions. Health Psychol 2008 May;27:379-387. [doi: 10.1037/0278-6133.27.3.379] [Medline: 18624603]
10. Sniehotta FF, Scholz U, Schwarzer R. Bridging the intention–behaviour gap: planning, self-efficacy, and action control in the adoption and maintenance of physical exercise. Psychol Health 2005 Apr;20:143-160. [doi: 10.1080/08870440512331317670]
11. American Psychological Association. APA Dictionary of Psychology - Self-Efficacy. 2022. URL: https://dictionary.apa.org/self-efficacy [accessed 2021-10-07].
12. Kok G, Gottlieb NH, Peters GJ, Mullen PD, Parcel GS, Ruiter RA, et al. A taxonomy of behaviour change methods: an intervention mapping approach. Health Psychol Rev 2016 Sep;10:297-312 [FREE Full text] [doi: 10.1080/17437199.2015.1077155] [Medline: 26262912]
13. Johnston M, Carey RN, Connell Bohlen LE, Johnston DW, Rothman AJ, de Bruin M, et al. Development of an online tool for linking behavior change techniques and mechanisms of action based on triangulation of findings from literature synthesis and expert consensus. Transl Behav Med 2021 May 25;11:1049-1065 [FREE Full text] [doi: 10.1093/tbm/ibaa050] [Medline: 32749460]
14. Williams SL, French DP. What are the most effective intervention techniques for changing physical activity self-efficacy and physical activity behaviour--and are they the same? Health Educ Res 2011 Apr;26:308-322. [doi: 10.1093/her/cyr005] [Medline: 21321008]
15. Olander EK, Fletcher H, Williams S, Atkinson L, Turner A, French DP. What are the most effective techniques in changing obese individuals' physical activity self-efficacy and behaviour: a systematic review and meta-analysis. Int J Behav Nutr Phys Act 2013 Mar 03;10:29 [FREE Full text] [doi: 10.1186/1479-5868-10-29] [Medline: 23452345]

16. American Psychological Association. APA Dictionary of Psychology - Knowlegde. 2022. URL: https://dictionary.apa.org/knowledge [accessed 2021-10-07].
17. Paek HJ, Hove T. Risk Perceptions and Risk Characteristics. Oxford, UK: Oxford University Press; 2017.
18. Lippke S. Outcome expectation. In: Zeigler-Hill V, Shackelford TK, editors. Encyclopedia of Personality and Individual Differences. Cham, Switzerland: Springer; 2017:3379-3381.
19. Carey RN, Connell LE, Johnston M, Rothman AJ, de Bruin M, Kelly MP, et al. Behavior change techniques and their mechanisms of action: a synthesis of links described in published intervention literature. Ann Behav Med 2019 Jul 17;53:693-707 [FREE Full text] [doi: 10.1093/abm/kay078] [Medline: 30304386]
20. American Psychological Association. APA Dictionary of Psychology - Social Support. 2022. URL: https://dictionary.apa.org/social-support [accessed 2021-10-07].
21. Sniehotta FF, Schwarzer R, Scholz U, Schüz B. Action planning and coping planning for long-term lifestyle change: theory and assessment. Eur J Soc Psychol 2005 Jul;35:565-576. [doi: 10.1002/ejsp.258]
22. Howlett N, Trivedi D, Troop NA, Chater AM. Are physical activity interventions for healthy inactive adults effective in promoting behavior change and maintenance, and which behavior change techniques are effective? A systematic review and meta-analysis. Transl Behav Med 2019 Jan 01;9:147-157 [FREE Full text] [doi: 10.1093/tbm/iby010] [Medline: 29506209]
23. Norman P, Conner M. Health behavior. In: Reference Module in Neuroscience and Biobehavioral Psychology. Amsterdam, The Netherlands: Elsevier; 2017.
24. Sam N. Affect Regulation. Psychology Dictionary. 2013. URL: https://psychologydictionary.org/affect-regulation/ [accessed 2021-10-07].
25. Larimer ME, Palmer RS, Marlatt GA. Relapse prevention. An overview of Marlatt's cognitive-behavioral model. Alcohol Res Health 1999;23:151-160 [FREE Full text] [Medline: 10890810]
26. Marlatt GA, Donovan DM. Relapse Prevention: Maintenance Strategies in the Treatment of Addictive Behaviors. 2nd edition. New York, NY, USA: Guilford press; 2005.
27. American Psychological Association. APA Dictionary of Psychology - Habit. 2022. URL: https://dictionary.apa.org/habit [accessed 2021-10-07].
28. Kwasnicka D, Dombrowski SU, White M, Sniehotta F. Theoretical explanations for maintenance of behaviour change: a systematic review of behaviour theories. Health Psychol Rev 2016 Sep;10:277-296 [FREE Full text] [doi: 10.1080/17437199.2016.1151372] [Medline: 26854092]
29. American Psychological Association. APA Dictionary of Psychology - Social Influence. 2022. URL: https://dictionary.apa.org/social-influence [accessed 2021-10-07].
