# Supplementary material for: A Digital Lifestyle Coach (E-Supporter 1.0) to Support People With Type 2 Diabetes: Participatory Development Study
Source: JMIR Hum Factors. 2023 Jan 12;10:e40017. doi: 10.2196/40017 (PMC9947918; doi:10.2196/40017)
Supplement: Multimedia Appendix 4 [file humanfactors_v10i1e40017_app4.docx]

**Multimedia Appendix 4. E-Supporter Psychological Exercises**

Table S1. Psychological exercises per determinant of behavior

| **Determinant** | **Nr** | **Name exercise** | **Content** |
| --- | --- | --- | --- |
| Motivation | 1 | Importance ruler | Participants are asked to indicate how important the health behavior is for them, and think about why this is the case. |
|  | 2 | Your values | Participants are asked to choose 2 values (e.g. happiness, health, family, success) and are then prompted to describe the relationship between their chosen values and the health behavior they focus on. |
|  | 3 | Look into the future | Participants are prompted to think about advantages and disadvantages of engaging in the respective health behavior over 5 years and then chose their most important advantage/benefit. |
| Self-Efficacy | 1 | Confidence ruler | Participants are asked to report in how far they feel confidence to engage in the health behavior, followed by the question why they believe this to be the case. |
|  | 2 | Personal strengths | Participants are prompted to choose two personal strengths (e.g., trustworthiness, perseverance) and think about how their strengths can help them to engage in the health behavior. |
|  | 3 | Looking back on past success | Participants are stimulated to look back on past successes of something they experienced to be difficult, including what they did and how they felt. |
|  | 4 | Imagine… | Participants are asked to image themselves 5 years from the present moment and write an e-mail elaborating on how they managed to engage in the health behavior and give themselves a compliment. Participants are offered the possibility to send this mail as a reminder |
|  | 5 | Problem-solving | Participants are prompted to think about different ideas on how to engage in the health behavior, then choose the best idea and indicate why this is the best idea |
| Planning | 1 | Choosing a mate | Participants think about a mate to support them in the health behavior and how they can support them |
|  | 2 | Dealing with tempting situations (coping planning) | Participants chose two situations which they experience to be tempting not to engage in the health behavior, and think of how to overcome or prevent these situations |
|  | 3 | Tell it to others | Participants are prompted to make a plan on how to tell others they plan to engage in the health behavior |
|  | 4 | Reward yourself | Participants are stimulated to think about when they would like to reward themselves, and how |
| Mood | 1 | Feeling better in your own skin | Participants are asked to plan something they enjoy, including when, how, with whom and think about the necessary preparations |
|  | 2 | Dwelling on nice things | Participants indicate three nice things they experiences within the last 24 hours |
|  | 3 | Write something nice to yourself | Participants are asked to write a nice note to themselves they can then receive two weeks later |
|  | 4 | Friends for life | Participants are prompted to think about ways to make new friends, such as starting a new hobby or signing up at a local sports club |
|  | 5 | Doing something for another | Participants are asked to think about how to do something nice for someone else. This includes planning what, when and for whom |
|  | 6 | My ideal life | Participants are asked to described their ideal life over a certain amount of years |
|  | 7 | Setting small goals | Participants are prompted to set a goal thereby selecting small steps to take |
|  | 8 | Being clear | Participants are asked to describe a situation in which they would have liked to be more clear, and how they might react in an ideal situation in the future |
| Stress | 1 | Charge your energy | Participants are prompted to think about three activities/things that energize them and plan them in |
|  | 2 | Relaxing | Participants are redirected to a relaxing podcast (8 minutes) |
